# Nanocomposites Based on Iron Oxide and Carbonaceous Nanoparticles: From Synthesis to Their Biomedical Applications

**DOI:** 10.3390/ma17246127

**Published:** 2024-12-14

**Authors:** Mirela Văduva, Andreea Nila, Adelina Udrescu, Oana Cramariuc, Mihaela Baibarac

**Affiliations:** 1 National Institute of Materials Physics, Atomistilor Street, No 405 A, 077125 Magurele, Romania; adelina.udrescu@infim.ro (A.U.); barac@infim.ro (M.B.); 2 IT Center for Science and Technology, 25 No. Av. Radu Beller Str., 011702 Bucharest, Romania; oanacramariuc@yahoo.com

**Keywords:** Fe_3_O_4_, nanoparticles, carbon nanotubes, graphene oxide, reduced graphene oxide, composites, drug delivery, hyperthermia, anti-cancer therapy, bone tissue engineering

## Abstract

Nanocomposites based on Fe_3_O_4_ and carbonaceous nanoparticles (CNPs), including carbon nanotubes (CNTs) and graphene derivatives (graphene oxide (GO) and reduced graphene oxide (RGO)), such as Fe_3_O_4_@GO, Fe_3_O_4_@RGO, and Fe_3_O_4_@CNT, have demonstrated considerable potential in a number of health applications, including tissue regeneration and innovative cancer treatments such as hyperthermia (HT). This is due to their ability to transport drugs and generate localized heat under the influence of an alternating magnetic field on Fe_3_O_4_. Despite the promising potential of CNTs and graphene derivatives as drug delivery systems, their use in biological applications is hindered by challenges related to dispersion in physiological media and particle agglomeration. Hence, a solid foundation has been established for the integration of various synthesis techniques for these nanocomposites, with the wet co-precipitation method being the most prevalent. Moreover, the dimensions and morphology of the composite nanoparticles are directly correlated with the value of magnetic saturation, thus influencing the efficiency of the composite in drug delivery and other significant biomedical applications. The current demand for this type of material is related to the loading of a larger quantity of drugs within the hybrid structure of the carrier, with the objective of releasing this amount into the tumor cells. A second demand refers to the biocompatibility of the drug carrier and its capacity to permeate cell membranes, as well as the processes occurring within the drug carriers. The main objective of this paper is to review the synthesis methods used to prepare hybrids based on Fe_3_O_4_ and CNPs, such as GO, RGO, and CNTs, and to examinate their role in the formation of hybrid nanoparticles and the correlation between their morphology, the dimensions, and optical/magnetic properties.

## 1. Introduction

In recent times, there has been a shift in focus towards the replacement of traditional anticancer therapies, characterized by the utilization of large doses of drugs that have been associated with adverse effects on the quality of life of patients. This has resulted in the rise of the exploration of alternative therapies, including HT, which have demonstrated potential in addressing the limitations of conventional approaches.

The use of biocompatible compounds guarantees the targeted transportation of drugs and their gradual release, which can then be eliminated from the system via natural excretory pathways, particularly through urine. This has the effect of minimizing the impact of these compounds on the health of patients. HT is a therapeutic modality that involves the use of magnetic compounds, which can be regulated by an applied external magnetic field, to elevate the temperature of the tissue in which cancerous formations have been identified. The most prevalent magnetic nanoparticles (MNPs) are those of Fe_3_O_4_, which are obtained through co-precipitation of Fe oxide precursors. In conjunction with CNPs, they have demonstrated enhanced efficacy in drug delivery, attributable to the cell membrane permeation characteristic of CNTs.

Additionally, considerable interest is directed towards the development of tissue regeneration stimulation systems, which integrate hydroxyapatite (HA) and Fe_3_O_4_–CNP combinations, where CNPs may encompass CNTs, GO, RGO, or carbon quantum dots. The interest in materials such as Fe_3_O_4_, CNTs, or graphene and its derivatives stems from their distinctive properties, including magnetic character, high biocompatibility, low toxicity, and the availability to be grafted with different chemical groups through functionalization with acids (for CNTs and graphene derivatives) or polymers (chitosan (CS) and polyethylene glycol (PEG)).

Graphene–metal oxide nanoparticle composites are by far the most common hybrids synthesized by the hydrothermal method. In particular, metal oxide nanoparticles can provide a number of advantages to graphene–metal oxide nanoparticle hybrids, including higher capacitance, which depends on the nanoparticle structure, size, and crystallinity. Additionally, they can inhibit the agglomeration and restacking of graphene [1].

CNTs are one-dimensional carbonaceous nanomaterials in which several important features are combined. Represented by a graphene sheet rolled into a cylinder, CNTs are represented by a narrow diameter starting from 1 nm and a length from tens of nm to µm [2]. Due to their conductive properties, mechanical strength, high surface area, and tunable ability to modify their functional groups at the surface, CNTs have been identified as interesting candidates for biomedical applications [3]. In addition, their ability to penetrate the cell membrane and to bind different drugs due to their modified wall surface provides them the potential to be explored as drug carriers and co-agents in imaging studies for the diagnosis of different diseases, including the earlier detection of incipient cancers. Furthermore, drugs or other therapeutic agents can also be encapsulated inside CNTs via both covalent and noncovalent interactions [4]. Graphene is another interesting carbonaceous material of atomic thickness, based mainly on sp^2^ hybridized carbon atoms arranged in a honeycomb lattice structure. The interest in this two-dimensional material has grown over the years due to its outstanding reported properties, such as thermal stability, mechanical strength, high conductivity, and surface area. Furthermore, the possibility of modifying its surface by functionalization has added another dimension to its potential applications [5,6]. Graphene can be synthesized as a layer through CVD or obtained by exfoliating graphite to form GO, which is then reduced to obtain RGO [7,8]. The presence of numerous oxygen groups enables graphene and its derivatives to be readily dispersible in water or aqueous solvents, thereby making them suitable for utilization in physiological environments and combination with other compounds, such as magnetic oxides.

Fe_3_O_4_ is the most commonly utilized magnetic nanocomposite material for applications in magnetic resonance imaging (MRI), drug delivery, and HT. Fe_3_O_4_ exhibits distinctive characteristics, including superparamagnetism. When coupled with its high biocompatibility and low toxicity, these characteristics render it an appropriate choice for use in physiological environments. MRI is a valuable tool for medical investigation, particularly in the early diagnosis of various diseases. Its unique characteristics, including temporal and spatial resolution, minimal exposure to radiation, and rapid acquisition, contribute to its efficacy in this regard [9]. The hybrids, which were formed by combining Fe_3_O_4_ with CNPs, possess dual-functional characteristics, which makes them promising candidates for use in imaging and drug delivery [10,11,12]. In contrast to other review articles that make oblique references to the use of combinations of Fe_3_O_4_ with carbon nanostructures, the present work focuses on a specific and well-defined aspect: the synthesis methods used to obtain hybrids. It discusses both cases in which the co-precipitation of iron oxide precursors occurs in the presence of the carbon structure and cases in which both components, Fe_3_O_4_ and CNPs, are already synthesized and modified by grafting functional groups of interest for drug loading. The following section will present the general synthesis methods of hybrid materials based on Fe_3_O_4_ and CNTs, respectively, as well as Fe_3_O_4_ and graphene derivatives. The description will focus on the details that differentiate the various synthesis methods, as well as an analysis of the parameters that characterize the structures obtained through these methods. The following sections provide information on the efficacy of these structures in applications such as drug delivery, imaging for diagnosis and monitoring of drug transport, and as an adjuvant in HT therapy with an effect on the local destruction of cancer cells.

While other works focus mainly on the functionalization of CNTs and graphene with the objective of enhancing their biocompatibility [13], this study employs optical characterization techniques with the aim of assessing the functionalization process itself. Furthermore, the correlation between the synthesis methods and the observed performances of the resulting hybrids in the biomedical applications under consideration, namely drug delivery, HT diagnosis, and therapy, as well as tissue regeneration through the insertion of biomaterials that stimulate the bone cell growth process, is investigated.

The external context indicates a need for greater effort to identify optimal drug delivery systems. These platforms must provide a high load content and specificity in delivery to the damaged tissue, and they must be able to be entirely eliminated from the system after releasing the drug.

MNPs were widely tested due to their capacity to be controlled under an external magnetic field (AC operating field) and directed to the target site where the loaded drug is released. In order to facilitate the loading process, biocompatible MNPs are modified by coating polymers or the synthesis of composites/hybrids containing CNPs, including GO, RGO, CNTs, and others. Additionally, Fe_3_O_4_ nanoparticles (NPs) are a valuable tool for cell labeling and tracking in MRIs [14]. The necessity to extend the functionality of Fe_3_O_4_ regarding their application in drug delivery is fulfilled by incorporating them into a more complex structure, namely composites with CNPs. CNP-based nanomaterials, which are commonly used in various fields, have been proven to be effectively employed in biomedical applications, including tissue regeneration, anti-cancer treatments, HT, imaging, and similar approaches. They can be considered as alternatives to traditional treatments such as chemotherapy, radiotherapy, and surgical interventions [15,16,17].

The primary rationale for utilizing CNTs or graphene derivatives pertains to their extensive surface area and the functional groups that remain following the synthesis, reduction processes, or functionalization process. These functional groups facilitate chemical interactions and the formation of weak bonds, including π–π interactions, which are essential for drug loading. The functional groups with oxygen are actively involved in colloidal stabilization and participate in the H-bond formation. CNPs have the potential to bond with heavy metals, allowing them to be tracked by an MRI [18]. Additionally, they can traverse cell membranes, enabling the release of drugs within cells where they can remain for extended periods due to their high hydrophobicity [18]. Moreover, the CNPs help in charging the MNPs, increasing the citotoxicity of the hybrid structures, and, together with the coexistence of Fe_2_O_3_ and Fe_3_O_4_, they provide a local heat increase [19]. It has been reported that tumoral cells exhibit a high capacity for the incorporation of MNPs, which renders them more susceptible to citotoxicity [20].

Over the years, systems based on CNPs have also been tested in tissue regeneration in combination with HA, one of the most studied implant materials for the regeneration of traumatized bone tissue [21]. In light of these considerations, the synthesis of biocompatible materials such as biopolymers or composites with CNPs has been attempted with the objective of accelerating the healing process, not only in bone tissue but also in other tissues. This approach is supported by controlled drug delivery to the affected site. Consequently, in addition to biocompatibility, the material must fulfill a number of requirements, such as high drug loading/adsorption capacity and optimal prolonged targeted release at the lesion site [22]. Moreover, it has been shown that CNPs and MNPs can enhance the therapeutic efficacy of a specific drug, particularly when both are employed in combination [12,23,24,25,26]. Other researchers have explored the application of MNPs and graphene derivatives [27,28,29]. Each of the individual components possesses distinctive attributes that facilitate advantages in drug transportation and delivery and may also confer benefits in the imaging monitoring part of the drug’s effect [30].

When considered separately, the MNPs, operating under the influence of an external magnetic field, have been demonstrated to generate a localized heat increase within the tissue, which has been shown to contribute to the eradication of cancer cells. Even in the case of CNTs alone, the combination of functionalized CNTs (fCNTs) and HT led to a significant reduction in the size of breast tumors, especially in the early stages of cancer [31].

MNPs are capable of facilitating the targeted delivery of drugs, enabling their controlled release and monitoring throughout the delivery process. In addition, CNTs functionalized with carboxyl groups allow easier crossing of the cell membrane, enabling drug delivery directly to the target. Although CNTs are biocompatible, the main limitation of CNTs remains their difficult metabolization, and by accumulating at a higher percentage, the CNTs become toxic for the cells or for the body, especially in the case of nonfunctionalized ones [32]. Moreover, according to Ding, L. et al. [33], the evaluation of the cytotoxicity of multi-walled carbon nanotubes (MWCNTs) on human fibroblast cells revealed the relationship between dose and gene expression effect, so that at a concentration of 0.6 µg/mL CNTs seriously affected the cellular functions of maintenance, growth, and differentiation [33].

One disadvantage of Fe_3_O_4_ is the low permeability of the cell membrane, which is the essential argumentation for the association of MNPs with different types of carbon structures such as CNTs and graphene. CNTs also have a good drug storage capacity by noncovalent binding to the carboxyl groups on the wall surface [34]. CNTs that are mixed with MNPs are treated with a mixture of acids both for their acid functionalization and for the purpose of shortening and opening the tubes. This facilitates the subsequent mixing or precipitation of MNPs in the presence of CNTs, which allows the MNPs to enter the tubes. The resulting compound exhibits improved and extended properties.

The manuscript includes an introduction to the current context of using hybrid nanoparticles based on Fe_3_O_4_ and CNPs in biomedical applications. It also provides details about the main synthesis methods used for their preparation, as well as results of optical and magnetic characterization correlated with important parameters (nanoparticle diameter, Ms, and SAR) that reveal their efficiency in biomedical applications. Furthermore, it reports on the results of studies on this type of material in drug delivery, HT, the combination of HT with chemotherapy, and novelties in BTE. In the end, the paper identifies the research gaps and future goals in the field and presents a summary of the main conclusions detached from the manuscript.

The current limitations of the materials discussed relate to their biocompatibility and dispersion in the physiological environment. These shortcomings can be solved by polymer wrapping (polymers such as PEG or its natural alternative and CS), the degree of drug loading, which can be solved by double functionalization of CNPs (CNTs, addressed here), the efficiency of drug release as a function of pH value (favored by acidic environment), and other important aspects that directly influence the performance of the synthesized hybrid materials.

## 2. Synthesis of Nanostructured Materials Based on Fe_3_O_4_ and Carbonaceous Nanoparticles

Prior to an in-depth examination of the synthesis methods of composites based on carbonaceous@Fe_3_O_4_ materials employed in the medical field, a comprehensive analysis of the impact of synthesis parameters on the structure and morphology of the aforementioned composites is presented. Additionally, an investigation of the functionalization process of carbonaceous materials, exemplified by CNTs, is conducted to ascertain its potential enhancement of the studied composite performance in biomedical applications.

The generation of numerous negatively charged carboxyl functional groups results in the intervention of iron ions in the system by electrostatic positioning at these negative centers, thereby serving as nucleation centers, where Fe^3+^ is found on the surface of the walls of fCNTs [35]. It has been demonstrated that the structure of CNT composites can be changed by modifying some synthesis parameters, including the duration of acid exposure and solvothermal treatment, the ratio of ferric precursors (Fe^3+^ and Fe^2+^), and the ratio of ethylene glycol (EG) to diethylene glycol (DEG). During the solvothermal process, the Fe^3+^ centers are reduced to Fe^2+^, which can then be co-precipitated with the formation of Fe_3_O_4_ nanocrystallites.

The formation of dipolar interactions between magnetic nanocrystallites exhibiting oriented aggregation, some dipolar interactions are formed, which can be negatively influenced by a high degree of viscosity of the nonaqueous phase [35]. As previously mentioned, changing the synthesis parameters may result in the formation of Fe_3_O_4_/CNT composites with different morphologies. Xiao, D. et al. observed that a necklace-like nanostructure with Fe_3_O_4_ islands on MWCNTs is formed upon 6 h exposure to an acid mixture treatment. However, upon longer treatment of 14 h, mesh-like nanostructures are formed in which the Fe_3_O_4_ spheres act as nodes (joints) connected with short MWCNTs [35]. In addition, both the ferric precursor and the EG, in accordance with the DEG, play a significant role in the formation of magnetic MWCNTs.

A study by Cao et al. [36] indicates that a third agent is required for the complete reduction of Fe^3+^ to Fe^2+^ with co-precipitation in Fe_3_O_4_, and this is due to the change in alkalinity of the medium. The hydrolysis rate of ferric chloride (FeCl_3_) can be increased to facilitate the formation of larger Fe_3_O_4_ crystallites by increasing the alkalinity [37]. As posited by Xuan, S. et al. (2009) [38], EG serves the dual function of both reducing agent and reaction solvent in the Fe_3_O_4_ formation process. Furthermore, the ratio of EG to DEG is also a tool to control the size of the crystallites that are desired to be synthesized.

CNTs may have several applications in imaging investigations as evidenced by previous research [39,40]. Through functionalization, they can serve as MRI contrast agents [41], radiotracers [42], chemical and biological sensors, bioactive agents in drug delivery, and as a supporting matrix. This is due to their optical properties, which can be detected by luminescence, fluorescence, and Raman scattering, as well as their electrical properties that allow for conductivity. Additionally, their large active surface area and ability to immobilize DNA structures [43] or other proteins [44] contribute to their versatility.

The use of CNTs in certain applications, such as in drug delivery and as a tracking agent in imagistic investigations, is currently constrained by a few limitations regarding their interaction with the drug during the loading process and dispersibility in a physiological environment. These issues can be solved by the double functionalization of CNTs, a process that is achieved through a two-step process. Initially, acids are employed to treat CNTs, resulting in the formation of a carboxyl group on the nanotube surface. Subsequently, the aromatic cycloalkene-cyclostructures facilitate the binding of a drug through a cycloaddition reaction [45,46]. A concrete example is the 1,3 dipolar cycloaddition, which involves the introduction of two orthogonal amino groups that are subsequently employed in the generation of double-functionalized CNT structures [46]. The functionalization process of CNTs is of significant importance, as evidenced by numerous studies [47,48] indicating that fCNTs can be directly transported into the cytoplasm of mammary cells. The biocompatibility of CNTs within the body has been evaluated on laboratory mice, and the results demonstrate that they neither induce cell death (by apoptosis) nor elicit immune reactivity by alerting immunocytes (lymphocytes) [49]. Another study was conducted to assess the biocompatibility of simple and fCNTs over a 2-year period, monitoring the subjects in whose bodies they were introduced. The study by Muller and colleagues revealed that the use of CNTs with short lengths in the µm range did not result in an increased risk of mesothelioma [50]. Moreover, the hypothesis that the functionalization of CNTs results in a reduction in their toxicity was supported by a study conducted by Sayes and colleagues [51]. This study demonstrated that the toxicity of CNTs is inversely proportional to the degree of functionalization. The administration of high doses of MWCNTs (20 mg/kg) did not result in any observable abnormalities 24 h after injection. Conversely, despite their high cell membrane penetration capacity and intrinsic magnetic properties, the dual nature of the components (semiconducting and conducting) and the differing anisotropy to external magnetic field action (maximum susceptibility value when the magnetic field intensity vector is oriented parallel to the CNTs structure) render CNTs ineffective when used alone in imaging and HT applications. In order to achieve optimal efficiency, it is essential to ensure a precise alignment of the CNTs, which can only be achieved in magnetic fields exceeding 10 T [52]. When functionalizing, a significant category of carbon nanoparticles is the functionalization with polymers, such as PEG. This process not only enhances colloidal stability but also reduces cytotoxicity in vitro, which is of particular relevance for graphene derivatives [53].

Another key challenge and current limitation in biological applications of CNPs, particularly graphene compounds, is the dispersion of the material in the solution. The objective is to synthesize water-soluble graphene nanosheets without the necessity of additional stabilizing agents. The stability is due to electrostatic repulsion between the negatively charged graphene sheets [54,55].

The large specific surface area of graphene alone (~2630 m^2^/g) suggests that it may have potential as a drug carrier. The most successful combination is represented by the combination of graphene with Fe_3_O_4_ in targeted and controlled drug delivery due to the significant contribution of MNPs that improve the speed and precision of drug delivery and even influence the cell response to drug contact by local temperature increase. The local heating effect is a consequence of the applied external magnetic field, which is a result of Brownian agitation. In addition to Fe_3_O_4_ and graphene, CNTs, in synergy with the previously mentioned materials, facilitate enhanced drug transport across the cell membrane, while fCNTs show a high degree of permeability at the cellular level due to their structure like the lipoprotein wall structure [1].

The most frequently employed methods for synthesizing magnetic composites based on CNP (GO, RGO, and CNTs) and Fe_3_O_4,_ are wet methods, namely: chemical blending [56,57], chemical precipitation [20] or chemical co-precipitation [58,59,60,61], solvothermal [62,63], heterocoagulation [64], wet chemical synthesis/thermal reduction method [65], microwave hydrothermal method [66], and, in the case of CNT/Fe_3_O_4_, co-precipitation [23].

### 2.1. Chemical Blending

In order to obtain GO–Fe_3_O_4_ composites through chemical blending, 20 mg of Fe_3_O_4_ nanoparticles were dispersed in distilled water at a concentration of 0.5 mg/mL and subjected to ultrasonication until a homogeneous dispersion was achieved. Subsequently, GO (20 mg) was exfoliated in H_2_O (60 mL) via ultrasonication, resulting in a homogeneous GO suspension. Subsequently, the carboxylic groups on the GO nanoflakes surface were activated through the use of N-hydroxysuccinimide (NHS; 8 mg) and 1-(3-dimethylaminopropyl)-3-ethylcarbodiimide (EDC; 10 mg). The prepared mixture of modified Fe_3_O_4_ and GO was stirred for a period of 2 h. The resulting GO–Fe_3_O_4_ hybrid nanoparticles were subjected to centrifugation, followed by multiple washes with water and ethanol. Subsequently, the samples were then dried at a temperature of 100 °C [56,57].

### 2.2. Precipitation and Co-Precipitation Synthesis Method

The synthesis of composites via precipitation or co-precipitation involved the combination of 20 mL of concentrated GO with a mixture of iron ion precursors in a 3:1 mass ratio, solubilized in 50 mL of deionized (DI) water. The iron ion precursors employed were FeCl_2_·4H_2_O and FeCl_3_·6H_2_O. The solution was maintained at a constant temperature of approximately 70 °C and mechanically stirred for 15 min. Subsequently, 4 mL of ammonium hydroxide (NH_4_OH) were added under stirring to achieve a pH of 10. The solution was then stirred and maintained at a constant temperature of 70 °C for a further 2 h. The resulting black precipitate (GO/Fe_3_O_4_), obtained by magnetic sedimentation, was then washed to neutral with DI water and dried overnight in a vacuum drying oven at 45 °C [20,58,59,60,61].

In the synthesis of nanocomposites based on Fe_3_O_4_ and RGO via a chemical co-precipitation procedure, 50 mg of GO powder were suspended in 100 mL of DI water by ultra-sonication for 30 min. Thereafter, the suspension was mixed with FeCl_3_·6H_2_O (0.2 g) and FeCl_2_·4H_2_O (0.1 g). Subsequently, the reaction mixture was purged with N_2_ gas to remove any residual dissolved oxygen, and it was then stirred for an hour. A solution of 15 mL of 8 M NH_4_OH was added dropwise to the mixture in order to precipitate the ferrous and ferric ions. Subsequently, hydrazine hydrate (1 mL of 70% *w*/*w*) was added to the mixture, and the reaction was conducted at 60 °C for 2 h under magnetic stirring. The Fe_3_O_4_–RGO product was collected by magnetic separation, washed several times with water/ethanol, and dried under a vacuum at 60 °C. Additionally, bare Fe_3_O_4_ NPs were synthesized by the same method, but without the addition of GO [61].

### 2.3. Solvothermal Synthesis Method

In the solvothermal process, two solutions are prepared. The initial solution contains amine-modified graphene (G–NH_2_) (20 mg), FeCl_3_·6H_2_O (270 mg), sodium acrylate (750 mg), and sodium acetate (750 mg). The second solution contains EG (0.5 mL) and DEG (9.5 mL). The two solutions were then combined to create a homogeneous solution, which was subsequently transferred to a stainless-steel autoclave for storage in a furnace at 200 °C for 12 h. The resulting solution, designated MG–NH_2_, was then rinsed repeatedly with water and an ethanol solution and finally dried under vacuum for 15 h [62,63].

The synthesis of composites can be broadly divided into two main steps: (i) The first step implies the preparation of the MNPs, which is typically achieved through co-precipitation from FeCl_3_·4H_2_O and FeCl_2_·2H_2_O, in a 3:1 or 4:1 ratio, Fe^2+^ to Fe^3+^. The oxidation instability of Fe^2+^ results in a significant conversion of Fe^2+^ to Fe^3+^ during the synthesis process. The precipitation occurs with NH_4_OH; (ii) in the second step, the CNPs, which are often reported to be RGO, are synthesized following the Hummer-modified method, which involves the chemical exfoliation of graphite to GO and further thermal reduction. Alternatively, chemical agents such as citric acid and hydrazine hydrate [67], ascorbic acid [68], glucose [69], hydrazine [70], and others can be used. Besides the classical method [20,57,58,59,60,61,65,71,72,73], various alternative methods have been explored, such as ball milling [74], thermal decomposition [75], solvothermal method [62,63], microwave hydrothermal method [48], LbL self-assembly technique [76], heterocoagulation [64], and the supercritical method [19] (Table 1).

The synthesis type is particularly important in terms of the size of the Fe_3_O_4_ nano-crystallites and, therefore, the size of the resulting nanocomposite particles. This is further correlated with their efficiency in drug delivery and hyperthermic therapy (Table 1).

**Figure 1:**
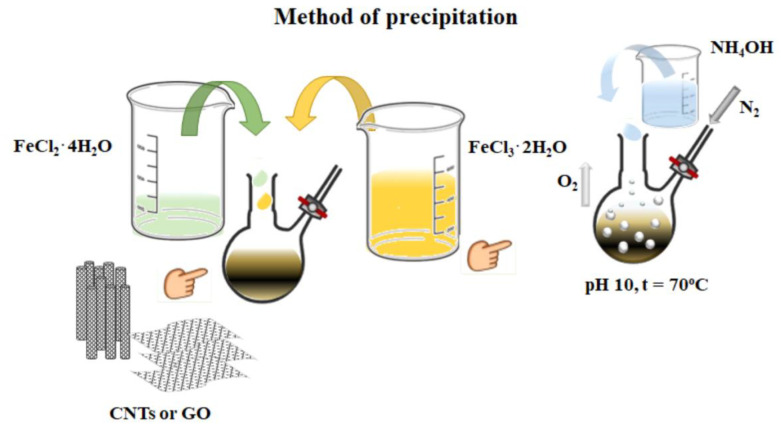
Method of precipitation used for the formation of composite based on Fe_3_O_4_ and CNPs, either CNTs or GO. Diagram created with Chemix (2024). Retrieved from https://chemix.org Accessed on 4 October 2024.

**Figure 2:**
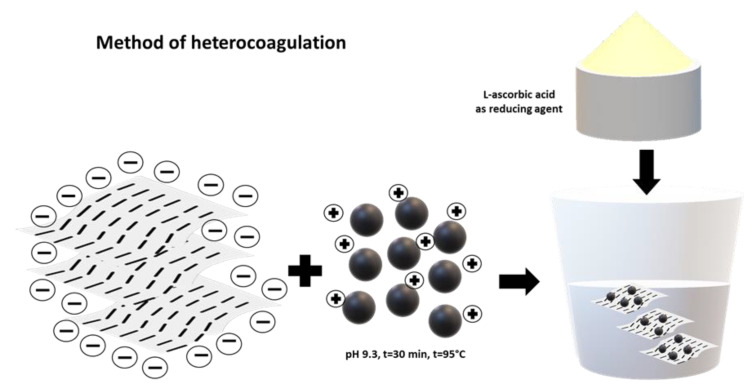
Method of heterocoagulation used for the formation of composite based on Fe_3_O_4_ and CNPs, either CNTs or GO.

The most representative methods used for the synthesis of composites based on Fe_3_O_4_ and CNPs, either CNTs or GO, are precipitation (Figure 1) and heterocoagulation (Figure 2). The first method differs from the simple chemical synthesis by introducing an additional step of in situ formation of Fe_3_O_4_, in the presence of GO, facilitated by the addition of NH_4_OH until a pH value of 10 is reached.

### 2.4. Heterocoagulation Synthesis Method

When it comes to heterocoagulation, the nanocomposites were prepared using oppositely charged GO and MNPs, at a pH ~5 as illustrated in Figure 2. This process simply involves the rapid mixing of the suspensions of GO and MNPs, which are subsequently kept under vigorous stirring for 15–20 min at room temperature. The reduction of composites was performed with L-ascorbic acid employing two distinct methodologies: an acidic approach at a pH ~3.5 and an alkaline approach at a pH ~9.3. The acidic conditions were maintained at room temperature, and a longer reaction time (4 days) was applied. In contrast, an elevated temperature (~95 °C) was used for 30 min in the alkaline medium [64].

### 2.5. Thermal Decomposition Method

In the case of other wet chemical synthesis routes, namely thermal reduction, specific amounts of FeCl_3_·6H_2_O, FeCl_2_·4H_2_O, and GO were weighed for each composition. The stoichiometric amounts of FeCl_3_·6H_2_O and FeCl_2_·4H_2_O were dissolved in DI water (25 mL) to obtain their 0.04 and 0.02 molar aqueous solutions, respectively. A dispersion of GO was prepared in DI water (250 mL H_2_O for 0.9 g GO) and subjected to sonication for 1 h to convert the carboxylic acid groups from the surface into carboxylate anions. Afterwards, a mixture of FeCl_2_·4H_2_O and FeCl_3_·6H_2_O (1:2 molar ratio) was dissolved in DI water, and it was added in a dropwise manner to the GO solution at room temperature under vigorous stirring. In order to facilitate the ion exchange reaction, a solution of aqueous NH_3_ (32%) was added in a gradual manner until the pH value reached 10, which is the required value for the formation of Fe_3_O_4_ NPs. Subsequently, the compositions were dried at room temperature (25 °C) [65].

### 2.6. Microwave Hydrothermal Synthesis Method

The samples were subjected to a 1-h sonication process during the microwave hydrothermal method, which was employed to facilitate the complete exfoliation of GO and its interaction with the iron hydroxide. The reduction process was conducted with hydrazine hydrate (1 mL), and the samples were then transferred to high-pressure microwave digestion vessels for elemental analysis. The samples were subsequently placed inside the microwave oven, which was operated at predetermined and optimized parameters; for example, 900 W, 250 psi, 200 °C, and 10 min. Subsequently, the samples were washed and dried, at 80 °C, for a period of 12 h [66].

The high frequency of use of a certain method for the preparation of these hybrid materials provides an indication of the most advantageous method for obtaining Fe_3_O_4_–CNP hybrid nanoparticles with reduced dimensions between 2 and 10 nm [19,20,23,24,41,57,58,59,60,61,62,63,64,65,71,72,73,74,75,76,77,78,79] (see Table 1 of the synthesis section). It can be stated that the most frequently used method is co-precipitation. The aforementioned advantages include the simplicity of the preparation process, the rapidity of the synthesis, the in situ preparation, the reduction of costs, and the capacity to control the dimension of the particles by modifying the synthesis parameters such as the quantity and concentration of the reactants and their ratio (in particular, the ratio between the precursors of Fe^3+^ and Fe^2+^).

### 2.7. Characterization of the Fe_3_O_4_/CNTs and Fe_3_O_4_/GO, Composites

#### 2.7.1. Optical and Morphostructural Characteristics

Optical and morphostructural characterizations are complementary and fundamental for the development of composites based on carbonaceous materials@Fe_3_O_4_. They guarantee the optimization of physicochemical properties, biocompatibility, and functionality, thus ensuring that they meet the specific requirements and the requisite level of rigor necessary for their biomedical applications. These characteristics are essential for the initial step of composite synthesis validation, as they significantly impact the subsequent magnetic properties. Once the morphostructural properties of the composites are first elucidated, the subsequent optimization of their optical properties can be achieved in a manner that meets the specific requirements of biomedical applications.

The interaction between Fe_3_O_4_ and carbonaceous materials can be illustrated through the use of Raman and FTIR spectroscopy, which corroborate the uniform distribution of Fe_3_O_4_ on the surface of carbonaceous materials. This is a crucial attribute for the optimization of magnetic properties. Furthermore, Raman and FTIR spectroscopy can be employed to identify the functionalization of composites with a range of agents selected to enhance dispersibility in physiological media (e.g., PEG and CS) or chemotherapeutic drugs (e.g., DOX) to facilitate the attachment and release of drugs in applications such as drug delivery and HT. In the realm of BTR, Raman spectroscopy can be employed to optimize the quality of carbonaceous materials in terms of defects, which in turn has a significant impact on the mechanical properties of the composite in bone structures. However, FTIR plays an essential role in identifying the biocompatible functional groups of the composite, which are crucial for osseointegration. Sometimes, additional XPS studies can be necessary to further understand the chemical surface of the composite that cannot be illustrated through Raman or FTIR.

The relevance of these characterization methods is supported by numerous studies in the literature (e.g., [19,23]), which demonstrate the impact of optical and morphostructural properties on the performance of carbon@Fe_3_O_4_ composites in biomedical applications. The following section presents a number of illustrative examples to demonstrate the significance of these analytical approaches.

The main interaction reported between the Fe_3_O_4_ and the RGO, GO or CNTs that were previously functionalized with carboxylic groups –COOH switched to anionic form –COO, often by following the ultra-sonication process in the alkaline medium, was the covalent bond confirmed through FTIR and Raman studies [58]. Among the measurements that specifically indicate the biomedical performance of the NPs involved in drug delivery [62] and HT [75], or even BTR [80], the hysteresis curves that provide information about the magnetic behavior of the samples recorded with vibrating sample magnetometer (VSM) and the specific absorption rate (SAR) of the magnetic nanocomposite under an applied magnetic field are the most common and important. There are also other important parameters, such as colloidal stability, which is considered in many reported studies [75]. Regarding the characterization of the hybrids in order to confirm the presence of both Fe_3_O_4_ and the carbonaceous compounds (GO, RGO, or CNTs), XRD, Raman scattering, AFM, SEM, TEM, and FTIR spectroscopy studies were performed.

**Figure 3:**
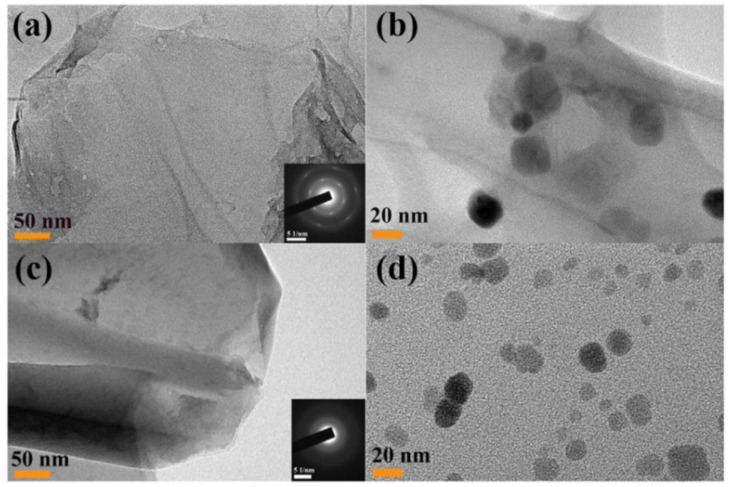
Low-magnification TEM images of (**a**) GO, (**b**) M-GO, (**c**) RGO, and (**d**) M-RGO [19]. Figure reused with permission from [19]. Copyright 2017 Elsevier.

The implementation of TEM analysis facilitates a comprehensive investigation of the nanoscale structure and properties of magnetite–graphene composites, which is pivotal for the advancement and improvement of these composite materials with enhanced performance. According to A. Tayyebi et al. [19], the TEM investigations depict the GO sheet with a wavy folded structure (Figure 3a) and the deposition of M (Fe_3_O_4_) nanoparticles on the GO surface via the co-precipitation method (Figure 3b). The image (Figure 3c) depicts RGO prepared in supercritical methanol with overlapped graphene layers, and with no distinctive pattern (SAED image), indicating an amorphous structure. Finally, the image (Figure 3d) depicts the Fe_3_O_4_–RGO nanocomposites, which exhibit smaller particle sizes and a more uniform distribution, with a notable reduction in agglomeration due to the supercritical conditions.

**Figure 4:**
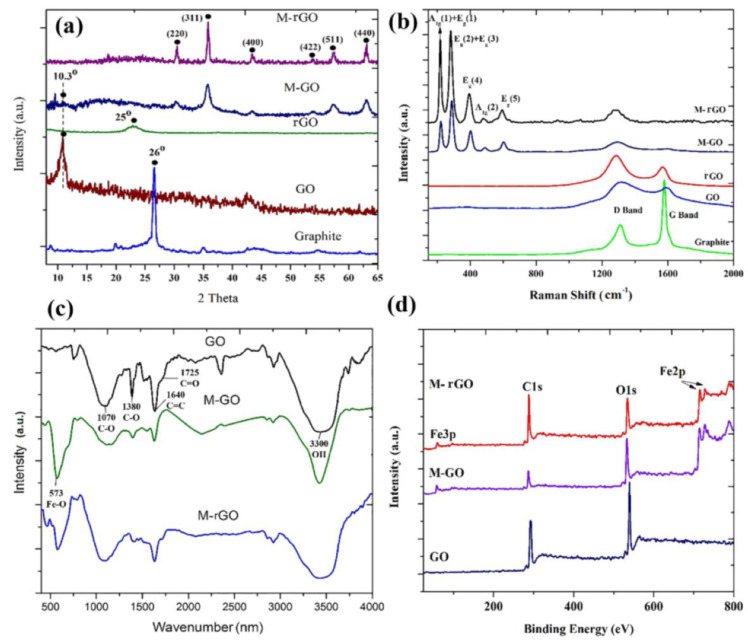
(**a**) XRD pattern, (**b**) Raman spectroscopy, (**c**) FTIR, and (**d**) XPS spectra of prepared materials [19]. Figure reused with permission from [19]. Copyright 2017 Elsevier.

In addition to TEM, XRD, Raman spectroscopy, FTIR, and XPS further investigations contribute to a deeper understanding of the characterization of the composite materials. Therefore, the XRD plot (Figure 4a) offers insight into the material structure and composition and demonstrates the stability of Fe_3_O_4_ on graphene structure in Fe_3_O_4_–GO and Fe_3_O_4_–RGO composites. The characteristic GO and RGO peaks demonstrate alterations in the interlayer spacing resulting from functionalization and reduction, whereas the Fe_3_O_4_ peaks substantiate their incorporation into the composite. Raman analysis (Figure 4b) offers insight into the internal structure and the manner in which the materials are integrated into the composite, which is crucial for an understanding of its functional properties. For instance, the D and G bands permit the evaluation of the degree of crystallinity and defects in the carbonaceous network for each material, and the presence of peaks characteristic of magnetite corroborates the attachment and stability of Fe_3_O_4_ nanoparticles in the Fe_3_O_4_–GO and Fe_3_O_4_–RGO composites. The FTIR spectra (Figure 4c) illustrate the distinctions between GO and its magnetite-functionalized derivatives, namely Fe_3_O_4_-GO and Fe_3_O_4_-RGO. The spectrum for GO displays the presence of multiple oxygenated groups, which are retained in the Fe_3_O_4_–GO spectrum, thereby confirming the integration of Fe_3_O_4_ into the composite. In the case of Fe_3_O_4_-RGO, the presence of Fe–O peaks and a reduction in oxygenated groups are observed, indicating an RGO structure with attached Fe_3_O_4_ nanoparticles. The XPS spectra presented in Figure 4d illustrate the variations in elemental composition between GO, Fe_3_O_4_-GO, and Fe_3_O_4_-RGO. The presence of C1 and O1 peaks serves to confirm the basic structure of oxidized graphene, while the Fe3p and Fe2p peaks indicate the integration and stability of Fe_3_O_4_ in the Fe_3_O_4_–GO and Fe_3_O_4_–RGO composites. The data indicate that Fe_3_O_4_ is effectively incorporated into the composites, resulting in a heterogeneous chemical composition that is conducive to applications that require the combination of graphene and magnetite properties.

The outer diameter of the fCNTs was in the range of 6 to 30 nm. The length of the CNTs may influence their dispersion in a solvent or in a polymer due to the fact that longer CNTs are prone to tangling. Distinct morphologies were observed as a consequence of different ratios between fCNTs and Fe_3_O_4_ (1:1, represented by H1, and 1:4 represented by H4) (see TEM images). When the ratio between the two components is at equilibrium (1:1), the Fe_3_O_4_ nanoparticles are mainly located on the surface of fCNTs in contrast to the composites with a higher percentage of Fe_3_O_4_ (4:1), where only a few crystallites are attached at the surface of fCNTs.

#### 2.7.2. Magnetic Characteristics

The review study published by Carlotta Pucci et al. [81] provides a comprehensive explanation of the existence of two types of iron oxide with a spinel structure and magnetic properties. The first refers to Fe_3_O_4_ (commonly referred to as magnetite), which is described as having a partially magnetized structure due to the intercalated coexistence of Fe^3+^ and Fe^2+^ ions. With the same crystal configuration in which only Fe^3+^ cations are distributed, having a complete magnetic character is the structure maghemite (γ-Fe_2_O_3_). As both forms are included when referring to magnetic iron oxide particles, it is necessary to recall the two forms, highlighting the similarities and differences between them, as well as the underlying reasons for their involvement in structures with biomedical applications.

In the aforementioned study, detailed insights are provided into the mechanisms by which alternating magnetic fields interact with magnetic iron oxide nanoparticles, functionalized either with biopolymers or in combination with other functionalized carbon-based structures for biomedical applications such as targeted drug delivery and localized tissue heating. As noted in the present work, the general conclusions of this comprehensive analysis highlight the positive influence of parameters such as nanoparticle size, dispersibility, magnetic saturation, and the frequency and amplitude of the alternating magnetic field on magnetic properties, including the specific absorption rate (SAR). Notably, higher frequencies and amplitudes are associated with increased SAR values. However, the power of the external magnetic field (calculated as the vector product of field intensity and frequency) must remain below a threshold of 5 × 10^8 ^A/ms to ensure compliance with safety standards and minimize the risk of adverse health effects for patients [81].

This study also explains why superparamagnetic particles are preferred over ferromagnetic ones, such as bulk ferric oxide. According to the research conducted by Vergés, M.A., and his collaborators [82], superparamagnetism is achieved at the nanoscale, and the application of a low magnetic field promotes the occurrence of amplified effects.

Iron oxide nanoparticles with diameters below the critical value of 30 nm exhibit superparamagnetic behavior [82,83], acting as single-domain magnets, in contrast to bulk oxides, which are characterized as multi-domain magnets.

Both magnetite and maghemite exhibit ferrimagnetic behavior, meaning they consist of two populations of atoms with antiparallel magnetic moments, similar to antiferromagnetism. However, the distinction lies in the dominance of one species, which results in a net magnetic moment that is different from zero [84]. When an external magnetic field is applied, all magnetic moments align in the direction of the field until the saturation magnetization is reached. Upon the removal of the external magnetic field, the magnetization does not completely vanish; a remanent magnetization (MR) persists. To return the material to its initial state, prior to the application of the magnetic field, an external field of a specific strength must be applied. This is referred to as the coercive field required to restore the material’s initial phase [82,83]. The orientation of the magnetic moment is not able to change instantaneously when an applied magnetic field is present. Instead, a delay in the magnetic response occurs. This phenomenon is exemplified by the hysteresis loop observed in the magnetic cycle [84,85].

Iron oxide nanoparticles (magnetite and maghemite) with sizes below the critical threshold of 30 nm have been observed to exhibit zero coercivity and remanent magnetization. However, superparamagnetic iron oxide nanoparticles (SPIONs) display high magnetic susceptibility, which enables them to be readily magnetized upon the application of an external magnetic field, in contrast to paramagnetic materials. When an alternating magnetic field is applied to SPIONs, the magnetic moments of individual particles (magnetic domains) align with the direction of the applied field, reaching magnetic saturation depending on the field direction.

Due to their magnetic properties, SPIONs can be oriented towards a specific area (such as the region affected by disease) through the application of an external magnetic field, ensuring high accumulation in the region of interest while diminishing the adverse effects. This approach has been demonstrated to result in enhanced effects when a drug is locally delivered [86,87].

One notable drawback of SPIONs is their tendency to aggregate due to van der Waals interactions between particles in suspension. This results in incompatibility with physiological fluids, particularly in aqueous environments [88]. The aggregation of SPIONs allows for the rise of magnetic dipole interactions between particles, which in turn affects their intrinsic magnetic properties [89]. Moreover, the formation of large aggregates results in immune system responses through recognition by the reticuloendothelial system (endoplasmic reticulum) [90]. This phenomenon was also highlighted by Joanna Dulińska-Litewka and her colleagues [91] and Vangijzegem, T. et al. [92], which indicates that SPION absorption by macrophage cells is facilitated. The advantages of SPIONs are numerous and include superior heating capacity compared to bulk ferromagnetic materials, as well as good dispersibility due to their zero remanent magnetization [81].

The topic of colloidal stability in aqueous environments has been widely addressed in numerous studies, including the work of Dulinska-Litewka et al. [91]. The proposed alternatives to overcome this issue encompass a diverse range of approaches, from treatment with acids (citric acid) [93] to coating with biocompatible, hydrophilic polymers such as PEG [94] or natural alternatives such as chitosan [95] or polysaccharides [96]. However, the colloidal stabilization of SPIONs through electrostatic repulsion remains less effective than steric stabilization achieved through polymer coating or functionalization with polymers [92].

Both in terms of enhancing biocompatibility through opsonization and improving the degree of dispersion to prevent particle agglomeration, literature reports suggest the use of polymer coatings for nanoparticles, with polymers such as PEG [97,98], PVA [92], polycaprolactone [99], and polyvinylpyrrolidone [100]. Additionally, other macromolecules, such as functional dendrimers (e.g., polyamidoamine (PAMAM)) [101] or peptides [102], may be employed. The specific polymer type exerts a significant influence on the nature of the interaction between the particles and the biological environment. This, in turn, has implications for the kinetics of SPION in the physiological environment [92].

Among the aforementioned polymers, PEG occupies a notable category. The coating of SPION nanoparticles with this polymer enables them to pass immune system tests and filters, as they are recognized as part of the system. The process of camouflage against the blood protein recognition system is referred to as opsonization. The compounds that actively participate in this process are known as opsonins, which protect SPION against adsorption and phagocytosis by macrophage cells [103].

In terms of organization, magnetite, and maghemite are composed of several magnetic domains, with sizes larger than 100 nm having been observed [103]. In contrast, for biomedical applications, the core size of these nanoparticles ranges from 5 to 60 nm, with a hydrodynamic dimension of 200 nm in the case of formulations used intravenously [91,104,105]. At such small sizes, the organization of the nanoparticles into magnetic domains is energetically unfavorable, resulting in the particles remaining in the singular domain state. The absence of magnetic domains is a defining characteristic of superparamagnetic behavior. Consequently, single-domain SPIONs are uniformly magnetized, with their magnetic moments aligning with the field when exposed to it. Superparamagnetic nanoparticles do not exhibit remanence or coercivity, and their magnetic moments vanish when the field action stops [106].

The term superparamagnetic is used to describe the behavior of SPIONs, which exhibit properties similar to those of paramagnetic substances but with a significantly higher magnitude. This is due to the fact that SPIONs with a single magnetic domain behave as a singular entity with a considerable magnetic moment. In the biomedical field, the superparamagnetic state is of significant importance for two reasons: Firstly, superparamagnetic nanoparticles do not retain residual magnetism after the removal of a magnetic field, thus avoiding the potential for clogging of blood vessels due to magnetic aggregation; secondly, each singular magnetic domain behaves as a giant magnetic moment, comprising the individual magnetic moments of the atoms that form the nanoparticle. This results in a high value of magnetic susceptibility [107].

Even though Fe_3_O_4_ has an excellent saturation magnetization [72,74], composites based on Fe_3_O_4_ and carbonaceous materials (such as CNT, GO, and RGO) are more frequently used in biomedical applications due to the increased biocompatibility resulting from coating Fe_3_O_4_ particles with these carbonaceous materials [57,61,108], which inhibit the reactive oxygen species (ROS) released by Fe_3_O_4_. Despite a diminution in magnetic properties due to the diamagnetic influence of carbonaceous materials in composites (M_s_ for Fe_3_O_4_ is 59.8 emu/g and respectively 48.9 emu/g for Fe_3_O_4_@RGO [72]), the remaining properties are sufficient for further investigation into their potential use in biomedical applications.

Moreover, carbonaceous materials present a very high specific surface area, thus having the ability to functionalize a large number of drugs, which can subsequently be used in processes such as drug delivery or hyperthermia. The magnetization of composites is influenced not only by the functionalization of Fe_3_O_4_ but also by the particle size of the composites themselves. For example, in Ref. [74], the authors observed a reduction in the saturated magnetization of the Fe_3_O_4_@GO composite, obtained by ball milling, from 78 emu/g (25 h of milling) to 42 emu/g (45 h of milling), which they attributed to the disordered magnetic moments of the particles with a reduced size. The smallest dimension of the nanocrystallites was reported for wet methods (precipitation, co-precipitation, and thermal decomposition) [71,72,75] under 10 nm. The size of crystallite is important because this parameter is correlated with the value reported for magnetic saturation, which increases at the small size of MNPs (Table 2).

The magnetization value of composites assumes great significance, as it serves to determine the response of the material to an external magnetic field and influence its potential applications. Additionally, the SAR value is a pivotal parameter for assessing the efficacy of the composites in transforming magnetic field energy into thermal energy, which is crucial for applications such as hyperthermia treatments. SAR is directly dependent on the magnetic properties of the composites and factors such as particle size and their interactions with the biological environment. Consequently, the correlation between magnetization and SAR represents an important avenue for optimization in the context of biomedical applications (Table 2).

Following this review of the magnetic properties of materials, we will examine a relevant example that illustrates these properties. This example comes from the study in Ref. [23], where the authors investigate the synthesis and characterization of Fe_3_O_4_@CNT composites. The study starts with a characterization of the composites in question by means of EM and XRD. In addition, the magnetic properties of the composites are evaluated.

**Figure 5:**
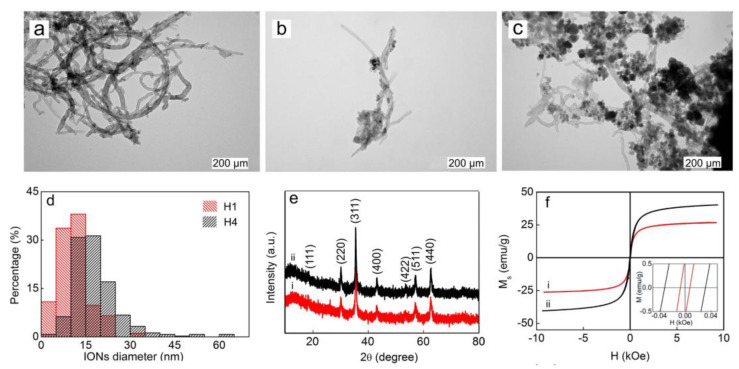
TEM microphotographs of fCNTs (**a**), H1 (**b**), and H4 (**c**) size distribution of Fe^2+^ and Fe^3+^ within hybrid materials (**d**); XRD patterns of H1 (i) and H4 (ii) (**e**); and the magnetic hysteresis loops of H1 (i) and H4 (ii) (**f**), where H1 represents 1:1 ratio of fCNTs: Fe_3_O_4_, while H4 is represented by 1:4 ratio of fCNTs: Fe_3_O_4_ (figure reused with permission from [23]). Copyright 2019 Elsevier.

Although both hybrids have the same phase composition, according to the XRD studies, the value of magnetic parameters differs significantly; namely, saturation magnetization was higher for H4 than for H1 (Ms H1 25 vs. Ms H4 43 emu/g), as well as remanence (Mr H1 0.4 vs. Mr H4 0.1 emu/g) and coercivity (Hc H1, 6 vs. Hc H4 30 Oe) [23]. A weak ferrimagnetic character was confirmed for both hybrids, H1 and H4, analyzing the hysteresis loops and the resulting magnetic parameters by comparison to bulk magnetite (Ms ~90 emu/g) (Figure 5).

The arrangement of iron ions, precursors of magnetite, at the surface of CNTs is influenced by the number and location of the negatively charged functional oxygen groups, which act as anchors for those cations, providing preferential reaction sites for ion co-precipitation. When there are not enough sites for co-precipitation to occur, the process of nucleation and growth seems to be initiated on the already formed magnetic particles, with the information available in the case of the H4 hybrid.

The analysis of all these data reported in the literature concludes that the magnetic properties of the Fe_3_O_4_–CNP hybrids are influenced by size, morphology, and composition. So, Świętek, M. et al. [23] reported that, after crossing the critical particle size of 16 nm (for Fe_3_O_4_ nanocrystallites), they become superparamagnetic and are characterized by a lack of remanence and coercivity. Meanwhile, in the absence of an external magnetic field, they behave as paramagnetic materials. While smaller particles were expected to be superparamagnetic, particles with a bigger diameter were ferrimagnetic. Accordingly, a higher value of Hc was reported for bigger particles when a higher amount of Fe_3_O_4_ was present within the hybrid structure (15–64 nm diameter) [23].

A comprehensive magnetic characterization of Fe_3_O_4_-based composites would greatly benefit from the evaluation of magnetic properties, which could be achieved by providing a range of macroscopic and microscopic data that is both complementary and informative. This approach would be of particular interest within the medical field.

In contrast to the macroscopic information provided by saturated magnetization, the EPR technique offers detailed insights into the dynamics of particles with unpaired electrons, which are specific to Fe_3_O_4_. Furthermore, it enables the investigation of microscopic interactions that are crucial for a comprehensive understanding of the changes induced by chemical and biological interactions [109]. Consequently, EPR could supplement conventional magnetic measurements, such as magnetization, by elucidating the magnetic properties of the magnetite nucleus (including spin interactions and magnetic relaxations), thereby facilitating a more profound comprehension of magnetic behavior under conditions pertinent to the targeted applications.

When the objective is to focus on biomedical applications, the immediate consideration is the potential for simple or more complex interactions with physiological and biological environments. In the utilization of sophisticated materials, such as Fe_3_O_4_@carbonaceous composites, EPR is capable of analyzing paramagnetic centers, including defects or free radicals, which may be implicated in a multitude of redox reactions, thereby generating ROS species. It is, therefore, crucial to assess their interaction with experimental environments, with the primary objective being the evaluation of oxidative stress, which plays a pivotal role in cell biology research and a range of biomedical applications [109,110,111].

In the field of structural biology, EPR spectroscopy can be employed to identify structural alterations in Fe_3_O_4_ when it interacts with serum components, including proteins and blood lipids [110]. A recent study (2024) has provided confirmation that EPR is capable of evaluating the signals emanating from the magnetic nuclei of nanoparticles and monitoring their behavior in biological fluids, such as blood, in order to ascertain their stability and biocompatibility [112].

Although EPR is not a commonly employed technique in the current literature on Fe_3_O_4_@carbonaceous materials composites, it offers a valuable opportunity to gain further insight into the interactions with physiological or biological environments. This facilitates a more profound comprehension of the manner in which these composites interact with their surrounding environment. Therefore, future strategies for the development of multifunctional materials with superior performance should employ EPR.

## 3. Applications for Hybrids Based on Iron Oxides and CNPs in the Health Field

The field of nanomedicine has witnessed considerable advancement over time, with the study and application of nanoparticles in diverse domains and novel therapeutic modalities. This is largely attributed to the superior characteristics exhibited by nanoparticles in comparison to their bulk counterparts. Among the materials employed in nanomedicine, Fe_3_O_4_ has emerged as a prominent choice due to its superparamagnetic or ferromagnetic properties. Currently, a multitude of Fe_3_O_4_-based devices have been deployed and validated in biomedical applications. The similarity in size between nanoparticles and biological molecules allows them to be used for both in vivo and in vitro research and applications. However, ongoing challenges for nanomedicine include understanding the long-term effects of the Fe_3_O_4_ material and its toxicity in living cells. The intracellular toxicity of pristine Fe_3_O_4_ appears to be mainly due to the production of ROS resulting from reactions of Fe ions with hydrogen peroxide formed by the mitochondrial respiratory chain from superoxides. These are known as Fenton reactions, from which H_2_O_2_ is transformed in more dangerous reaction products: (I) Fe^2+^ + H_2_O_2_ → Fe^3+^ + HO∙ + HO^−^; (II) Fe^3+^ + H_2_O_2_ → Fe^2+^ + HOO∙ + H^+^ and (III) 2H_2_O_2_ → HO∙ + H_2_O [113,114]. ROS species, such as HO∙, HOO∙, and H^+^, can be harmful in living cells as they may be involved in undesirable biochemical reactions.

Oxidative stress occurs when there is an excess of ROS species that surpasses the detoxification mechanism and the capacity of antioxidants to degrade them. This can cause significant harm to living cells and result in damage to proteins, lipids, and DNA. In fact, an increased concentration of Fe_3_O_4_ nanoparticles used in HT is cytotoxic in biological environments. For instance, in the in vitro study reported by Xie Y. et al., the authors demonstrated that the cytotoxicity of Fe_3_O_4_ on living cells is dependent on their nanoscale size [115]. The researchers observed a loss of cell membrane integrity for particles of 14 nm in size and mitochondrial dysfunction in hepatoma SK-Hep-1 and Hep3B cells for particles of 9–10 nm in size. This was attributed to the intracellular accumulation of ROS species, which ultimately led to cell necrosis. The cytotoxicity effects of Fe_3_O_4_ nanoparticles with varying sizes (60, 120, and 250 nm) were also identified on chicken macrophage cells [116]. The authors determined that reducing particle size had a more pronounced impact on cell viability, oxidative index, ROS levels, apoptosis, and proinflammatory secretion. Furthermore, an in vivo study [117] corroborates the findings of the in vitro studies presented above. In this study, Fe_3_O_4_ solutions of varying particle sizes (10, 20, 30, and 40 nm) were administered intravenously to adult mice, and the resulting effects were observed. Although the hematological and histological analyses did not show significant toxicity, the smallest particles of Fe_3_O_4_ highlighted the presence of oxidative stress, which determined the change in the gene sensitivity level expression.

The above studies are based on the fact that as particles shrink in size, their surface area increases. These results lead to a greater percentage of their atoms/molecules being exposed on the surface, thereby facilitating their absorption within biological systems and enhancing their reactivity. Moreover, a considerable number of crystalloid discontinuities allow for the rise of a substantial number of defects that also function as reaction centers [114]. In summary, it is crucial to evaluate the particle sizes of the magnetic materials employed in biomedical applications from a toxicological standpoint. Alternatively, strategies for particle coatings may be essential to modify their surfaces.

Rigorously optimizing the surface particularities of Fe_3_O_4_ with a view to reducing cytotoxicity, inflammation, and immune response in living cells represents a significant and pressing challenge for medical clinics and demands careful and rigorous consideration. It is of paramount importance to regulate the coating strategies that modify the morphology of nanoparticles, their tendency to aggregate, and their surface charge to guarantee the optimal interaction of Fe_3_O_4_ with biological environments. Surface passivation of Fe_3_O_4_ with biocompatible materials reduces the long-term toxicity effects caused by the body’s immune response and improves cell activity and proliferation. PEG is a water-soluble polymer that is frequently employed in the modification of magnetic materials due to its ability to facilitate protein rejection, prevent immunogenicity, and exhibit high biodegradability in physiological environments. Despite the FDA’s approval of PEG as a safe and biocompatible material, the synthesis of its composites with Fe_3_O_4_ necessitates adherence to rigorous conditions, including elevated temperatures and a nitrogen atmosphere [118,119]. EPR has demonstrated that graphene-based materials, such as RGO, and GO, exhibit a potential antioxidant effect, protecting organic dyes from HO∙ and HOO∙ oxidation [120]. The studies highlight that the graphitic network serves as the primary active site for scavenging HO∙ and HOO∙, forming radical adducts at sp^2^ carbon centers, and protecting against ROS generation and oxidative stress. Therefore, modifying the Fe_3_O_4_ surface with graphene-based materials is a strategy that should be considered for protection against ROS.

Although graphene-family materials are considered toxic in either in vitro or in vivo media [61,121], their composites can be safely considered in HT procedures as they provide cell viability [57,108,121]. There are several studies that highlight the great biocompatibility of Fe_3_O_4_@graphene-based composites presented in Table 3.

**Figure 6:**
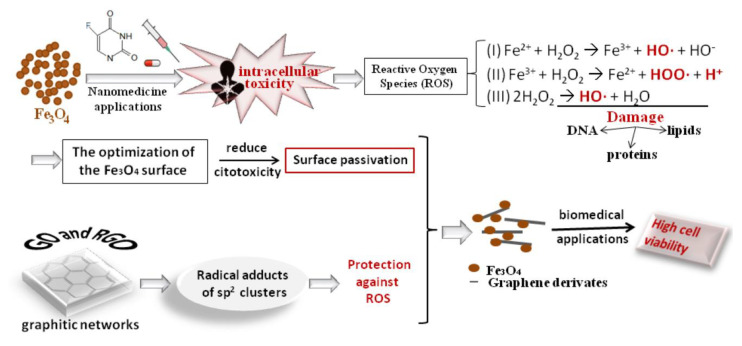
The optimization of the Fe_3_O_4_ surface with graphene derivatives to obtain a biocompatible composite capable of being used in cellular environments for biomedical applications. Diagram created with Chemix (2024). Retrieved from https://chemix.org. Accessed on 9 October 2024.

The passivation scheme of Fe_3_O_4_ with graphitic networks, which serves to protect against ROS, is illustrated in Figure 6. 

### 3.1. Applications in Drug Delivery

#### 3.1.1. Composites Based on Graphene-Fe_3_O_4_

The selection of graphene@Fe_3_O_4_ composites with favorable solubility and dispersibility in physiological and tumor environments represents a pivotal initial stage in the development of a high-performance drug delivery system.

In general, graphene exhibits hydrophobic tendencies as a typical consequence of π–π interactions between the benzene rings and the absence of hydrophilic functional groups containing oxygen. The inability to separate the graphene layers presents a challenge to achieving optimal solubility and dispersibility in aqueous solutions, consequently affecting its medical performance. Nevertheless, to the credit of the literature, there are two published studies that have investigated the potential of graphene nanosystems in magnetic drug delivery applications [122,123]. In both cases, the authors utilized cross-linked polymers that wrapped around graphene sheets, thereby facilitating the hydrophilic behavior of the composites used as drug delivery systems. Once this characteristic is achieved, it is anticipated that drug loading efficiency, a crucial parameter in drug delivery applications, will increase. This is due to the fact that the active surface area of a well-dispersed composite will become larger and more accessible for drug loading (Table 4).

In light of the numerous endeavors to optimize the effectiveness of graphene in drug delivery, it is evident that graphene derivatives, in particular, GO (sometimes even RGO), have attracted considerable interest due to the chemical surface of oxygen-containing groups, which facilitate graphene’s hydrophilic nature. Moreover, the negative effects of GO or RGO aggregation can be mitigated through their functionalization with polymers, which exhibit remarkable dispersibility in physiological or tumor media and serve as efficacious drug delivery vehicles, facilitating a high level of loading capacity for pharmaceutical agents. Consequently, many publications exist in the literature that attest to the integration of polymer-functionalized GO or RGO with MNPs (Fe_3_O_4_) for the purpose of drug delivery. Polyethylene glycol (PEG) is the most valuable biocompatible and nontoxic hydrophilic polymer used in drug delivery-based graphene anchoring, having received approval from the FDA. Despite the encouraging preliminary results [67,68] summarized in Table 5, the nonbiodegradable nature of PEG and the potential for adverse effects arising from its accumulation in physiological media have prompted the exploration of alternative polymers to modify the surface of graphene-based derivatives. CS is a natural polymer that has the potential to mimic biological systems and can therefore be used as an alternative to PEG. The researchers observed that graphene-based derivatives exhibited a high degree of dispersibility in magnetic drug delivery carriers developed by grafting the CS biopolymer onto the surface of RGO [63] or GO [76] through its functioning amino and hydroxyl groups, which are hydrophilic in nature. The mucoadhesive properties and polycationic nature of CS in acidic tumor media have been observed to facilitate enhanced penetration into cell membranes, as evidenced by a high drug loading efficiency [63,76] presented in Table 5. Derived from CS, GA is another polymer that has been successfully employed as a solubilizer and dispersant of GO, with the objective of improving the efficacy of the GO–GA@Fe_3_O_4_ drug delivery system [58].

A crucial aspect of drug loading is the choice of an appropriate carrier system, which should be made on the basis of an understanding of the nature of the system–drug interaction and the strength of this interaction. The loading efficiency is typically determined by quantifying the concentration of free drug using ultraviolet-visible (UV-VIS) spectroscopy. For instance, the optimal loading effectiveness of the anthracycline chemotherapeutic drug, specifically DOX, on the nanocomposite surface of GO@Fe_3_O_4_ is demonstrated by a maximum encapsulation efficiency of approximately 80% for a DOX concentration of 0.2 mg/mL [126]. However, M. Xie et al. [76] achieved enhanced dispersibility of the GO@Fe_3_O_4_ composite through the LbL self-assembly of GO magnetic nanosheets functionalized with CS and sodium alginate. This procedure results in enhanced exposure of the GO layers to the drug, thereby achieving a greater efficiency of up to 137% for a DOX concentration of 0.2 mg/mL. The high drug loading efficiencies are typically attributed to the extensive area of the GO surface, which allows for the incorporation of a significant quantity of pharmaceutical agents through functionalized graphene sheets. Additionally, the strong π–π stacking interactions between the aromatic rings of DOX and the entire sp^2^ hybridized honeycomb area of GO sheets contribute to the effective loading of the drug. Furthermore, H-bonds play a pivotal role in enhancing the drug loading efficiency, and they can be formed interchangeably between the strong electronegative atoms of DOX, represented by the -NH_2_ and -OH groups, as well as the hydrophilic functional groups of GO (-COOH and -OH). It can be reasonably presumed that, as a consequence of their aromatic molecular structure and the presence of hydrophilic groups (such as -NH_2_, -C=O, -OH, and -COOH) in their crystalline structure, the majority of chemotherapeutic drugs can be anchored upon the external surface of graphene-based materials by means of π–π stacking interactions and a variety of possible hydrogen bond configurations, depending on the presence of oxygen-containing chemical functional entities on the graphene surface. In addition to DOX [76,126], this phenomenon has also been observed in other pharmaceutics such as 5-FU [10,127,128], MTX [73,129], DNR [58], and PTXL [123]. All these publications imply the use of graphene@Fe_3_O_4_ nanocarriers for drug delivery with great loading efficiencies. After the drug loading process, the Fe_3_O_4_ nanoparticles, which are not coated with the chemotherapeutic agent, are permitted to move freely when an external magnetic stimulus is applied, thereby directing the drug to the target cell line (in vitro studies) or tumor (in vivo procedures) in the vicinity of which the magnet is preferably fixed. It is, therefore, evident why a nanocomposite containing both graphene derivatives and Fe_3_O_4_ nanoparticles is of great importance for effective drug delivery applications.

Once the anticancer drug has reached the vicinity of the cancer cells, the drug is released into the tumor environment with a high degree of efficiency, which is strongly dependent on the acidity of the environment. Due to the pH sensitivity of drug delivery platforms, in vitro experimental studies have thus comparatively highlighted their behavior in both acidic and basic environments. This has involved the simulation of a phosphate-buffered biofluid saline and the quantification of the amount of medicine by UV-VIS absorbance. For instance, a pH-sensitive behavior was observed in the case of DOX [45,74] and other drugs, including 5-FU [10,122] and MTX [129], as summarized in Table 6.

The findings of these studies are consistent and based on the protonation of the -NH_2_ groups of the drugs, which consequently weakens the hydrogen bonds formed between the drug and the conjugated graphene-based Fe_3_O_4_ nanocarriers. As a consequence of the observed pH dependence, anticancer drugs can be distributed in a specific manner around tumor cells, rather than being released nonselectively throughout the surrounding healthy tissues. This is a consequence of the lower pH microenvironment of cancer cells relative to healthy cells, which is a consequence of the increased lactic acid flux that cannot be transferred immediately to the blood vasculature [63,129,130].

Once the mechanisms of drug loading and release have been elucidated, it becomes pertinent to investigate the in vitro and/or in vivo cellular uptake of anticancer drugs from nanocarriers and their cytotoxicity in various cell lines. Cellular uptake is inherently inefficient in cell culture media. However, the process can be made highly effective when external assistance is provided via a magnetic field [131]. This is why composites containing Fe_3_O_4_ nanoparticles represent a significant advancement in the scientific community’s understanding of drug delivery. Following cellular uptake of pharmaceutical drugs, a pivotal question is whether this process influences tumor cell cytotoxicity without affecting healthy cells. The answer can be derived from an analysis of the existing literature up to 2024, as summarized in Table 7 below.

What is important about the last two studies [125,130] from Table 7 is that, although PEG anchoring in graphene-based magnetic drug delivery systems may potentially impede cellular uptake due to its shielding properties and accumulation in physiological media, the authors have demonstrated that the GO–PEG@Fe_3_O_4_ nanocarrier exhibited good internalization capability and no cytotoxicity effect in various cell lines (Table 7). Furthermore, beyond these in vitro studies, it was observed in 2012 [125] that the same composite exhibited high internalization of the 4T1 tumor in a real BALB/c mouse, accompanied by a cytotoxicity of approximately 67%. Also, in Ref. [63], V. Karthika et al. showed no toxicity of the RGO–CS@Fe_3_O_4_ nanocomposite in zebrafish embryos. It would appear that these will remain the only two in vivo tests of this nature until 2024, which involve the injection of a graphene@Fe_3_O_4_ composite into a living animal [125].

**Figure 7:**
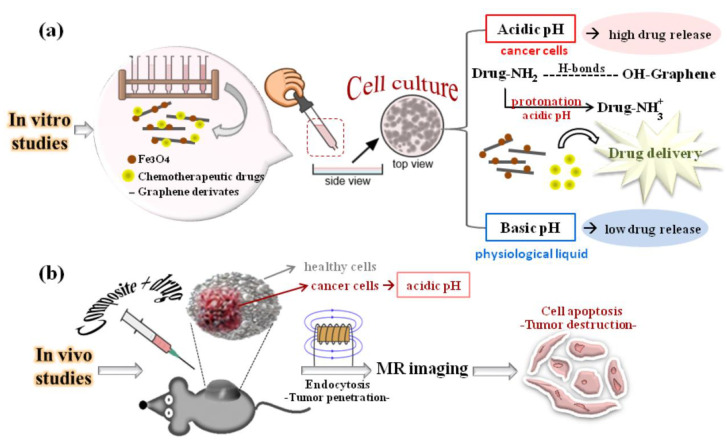
Diagram of the drug deliveries in both in vitro (**a**) and in vivo experimental studies (**b**) implying composites of graphene derivatives, Fe_3_O_4_ nanoparticles, and anthracycline c hemotherapeutic drugs. Diagram created with Chemix (2024). Retrieved from https://chemix.org. Accessed on 10 October 2024.

Based on the aforementioned observations, a model of the release mechanism from a carrier system comprising both conjugated graphene derivatives and Fe_3_O_4_ is presented in Figure 7.

#### 3.1.2. Composites Based on CNTs-Fe_3_O_4_

The employment of CNTs as drug delivery systems has been met with success, largely due to their capacity to facilitate controlled drug targeting, promote cellular uptake via endocytosis, and provide a large surface area with excellent drug attachment capacity. Similarly, the hydrophobic surface of CNTs enables cellular internalization and enhances susceptibility to π–π stacking interactions with pharmaceutical drugs that typically contain aromatic rings in their structure, in a manner analogous to graphene. Furthermore, the potential for straightforward functionalization makes them a promising choice for drug delivery operations including chemotherapy.

Despite its remarkable properties, the toxicity of pristine CNT, of the type SWCNTs or MWCNTs, and its solubility in physiological environments is a challenging issue that can be overcome by covalent or noncovalent functionalization with hydrophilic molecules or macromolecules. Several strategies exist for functionalizing CNTs in order for the drug delivery to increase selectivity, blood circulation time, or drug carrier capacity, in addition to solubilizing the CNTs and increasing their biocompatibility. For instance, researchers have successfully modified CNT surfaces with polysaccharides, such as CS or its derivates, using noncovalent bonds like H-bonds or hydrophobic interactions, and found a drug load efficiency of about 75% [132]. Covalently bonding PEG to CNTs is another strategy for improving their pharmacokinetic properties. This can result in a high level of drugs being delivered by a low dose of CNT, with a drug loading efficiency of up to 134,000 molecules [133].

The utilization of MNPs for the modification of CNT surfaces paves the way for novel avenues in nanobiomedicine, facilitating the preparation of efficacious nanohybrids as a means of developing groundbreaking drug delivery systems. In contrast to conventional methodologies, this approach facilitates the enhancement of drug targeting efficiency and trajectory manipulation in an external magnetically induced field. Furthermore, the most notable benefit of MNPs incorporated into these composites is their capacity to distinguish tumor cells from adjacent healthy tissue, thus minimizing the likelihood of adverse reactions related to the drug delivery process. The efficacy of CNT-based nanocomposites functionalized with Fe_3_O_4_ and biocompatibility-enhancing agents, such as micelles or dendritic copolymers, in advanced drug delivery systems has been demonstrated in previous studies [22,134]. These studies have shown that these nanocomposites can significantly enhance the cytotoxicity of drugs against cancer cells [22,134] and facilitate controlled drug release rates [22]. These findings are summarized in Table 8.

Although the number of in vitro studies on Fe_3_O_4_ composites functionalized with CNTs is limited, there is evidence that this approach has been extended to more comprehensive research. Due to these considerations, two relevant studies have explored the use of CNT@Fe_3_O_4_ composites, demonstrating their potential for use in advanced drug delivery nanoplatforms. These include complex in vivo/in vitro studies of lymph node metastasis [79] and malignant brain gliomas [78], which are presented in the following research investigations.

The utilization of liposomes, polymers, and micelles as drug carrier systems represents a prevalent methodology for facilitating lymphatic drug uptake [135]. Although these systems display favorable lymphotropic drug carrier characteristics, there is a necessity to enhance their tumor cell targeting efficiency in order to optimize chemotherapy effectiveness. This is the reason why the utilization of nanoparticles as targeting pharmaceutical delivery systems has been the subject of this investigation. Metastatic tumors usually spread through the lymphatic system in 80% of cases and may appear at an early stage of the disease. It is unfortunate that chemotherapeutic drugs administered orally or intravenously are poorly absorbed by the lymphatic tissue. This presents a significant challenge to the effective delivery of these drugs and, thus, a compelling rationale for the exploration of alternative drug delivery strategies that could enhance survival rates. Nanometer-sized particles can cross the lymphatic system during drug delivery procedures, but not all anticancer drugs can be transferred to the nearby lymph nodes. Nevertheless, the researchers have demonstrated that composites comprising CNTs and iron oxides can effectively deliver drugs to the lymphatic network, which is advantageous for the therapeutic management of lymphatic metastases that would otherwise remain unaddressed with current methodologies.

A schematic representation of the in vivo study utilizing a Fe_3_O_4_@CNT composite as a drug delivery vehicle in lymph node metastasis is presented in Table 9.

The drug delivery performance was evaluated by comparing the composite to a well-known drug carrier, namely a nanosized activated carbon system decorated with Fe_3_O_4_. As expected, the chemotherapeutic agent was preferentially absorbed by the lymph nodes with a maximum concentration of 6 µg/g for MWCNTs grafted with Fe_3_O_4_, compared to 3 µg/g for the activated carbon system. The combination of the magnetic effects of Fe_3_O_4_ with an externally imposed magnetic field results in enhanced lymph node retention, thereby facilitating more exact targeting of the drug and achieving values of 9 µg/g and 6 µg/g, respectively. This study demonstrates the high efficacy of the nanoplatform that uses CNTs as drug carriers, making it a promising candidate for lymphatic drug delivery, prohibition, or treatment of cancer metastasis with minimal side effects and no recurrence.

Glioma is a malignant cerebral tumor that occurs in 78% of individuals with brain cancer. Although surgery, radiotherapy, and chemotherapy treatments are available, their effectiveness can be limited due to the rapid invasion of the neural network and the unfavorable impact on the immune system. Despite the paucity of pharmaceuticals available for this ailment, the blood–brain barrier—a membrane separating the circulatory and nervous systems—limits the number of chemotherapeutic agents that can be introduced, rendering it challenging to attain the optimal dosage and consequently leading to an inadequate therapeutic response. However, nanoparticles such as CNT can effectively penetrate the blood–brain barrier, transport drugs, and deliver them to cancerous tumors [136]. Furthermore, in order to improve the therapeutic efficacy of drug delivery, it is essential to enhance both the targeting and the controlled drug release. This can be achieved by incorporating magnetic particles into the CNT matrix. A schematic representation of the in vitro study utilizing a Fe_3_O_4_@CNT composite as a drug delivery vehicle in malignant cerebral tumors is presented in Table 10.

To enhance targeting capabilities, the authors immobilized folic acid onto the composite surface, thereby increasing its affinity for the receptors of cancer cells. In fact, the authors developed a dual targeting system by combining the attraction of folic acid to cancer cells with the targeting of the magnetic composite in a magnetic field. Thus, the in vitro study demonstrates that anti-cancer DOX-loaded MWCNTs@Fe_3_O_4_ has a higher killing efficiency of the glioblastoma cells compared to free DOX, even with a reduced drug dose, due to the effective cellular internalization via endocytosis and the magnetic targeting effects. The performance of the folic acid-grafted MWCNTs@Fe_3_O_4_ drug delivery system was evaluated considering the drug loading efficiency and DOX release rate presented in Table 10.

These findings collectively illustrate the efficacy of the Fe_3_O_4_@MWCNTs magnetic drug delivery system in addressing glioma and offer a rationale for its prospective utilization in the management of other malignant conditions.

### 3.2. Applications in HT

Magnetic HT has been introduced in oncology as a noninvasive alternative or complementary treatment to chemotherapy and radiotherapy [20,72,137]. The mechanism involves overheating the local tumor tissue to a temperature range of 43–46 °C through the introduction of MNPs into the target cell within an extrinsic magnetic field that exhibits alternating polarities. When heated, the cancer cells allow the MNPs to penetrate them through endocytosis, raising the local temperature and inhibiting thermal energy dissipation [138]. Due to these circumstances, the degradation of cytoplasmic proteins and deterioration of the tumor vasculature structure at high temperatures prevent the delivery of nutrients and oxygen to the malignant cells. Depending on the temperature used, this phenomenon determines a cellular degradation that finally generates apoptosis (mild HT: 43 °C) or necrosis (thermal ablation: 46 °C) without affecting the highly vascularized healthy surrounding tissue. Depending on the heat dosage, tumor cells are either naturally eliminated from the organism or become more susceptible to destruction due to their oxygen destitution and damage to cytoplasmic proteins.

Magnetic iron oxides based on Fe^2+^ and Fe^3+^ are the only biocompatible MNPs used in derivatives such as Feraheme^®^, which were approved by the FDA for use in biological applications [139,140]. The most studied metal oxide in HT and already approved in medical and clinical applications is Fe_3_O_4_ [141,142] or Fe_3_O_4_ in composites with carbonaceous materials (GO or RGO) [19,65,66,74,75]. Fe_2_O_3_ pristine has undergone preliminary testing for potential utilization in HT [143,144], and was found to be susceptible to oxidation, a factor that requires urgent consideration. A review of the literature reveals no published studies on Fe_2_O_3_–carbonaceous composites for HT applications.

The initial demonstration of using magnetic particles for cancer treatment via HT was conducted by Gilchrist et al. in 1957 [145]. With the advancement of nanotechnology, MNPs have been widely used in HT research [141,143] not only because of their nontoxicity but also because of their magnetic properties and their multiple functionalization possibilities. However, the use of unmodified particles in this field is somewhat limited due to their tendency to agglomerate, which reduces their activity and the circulation time in the body. This inconvenience can be overcome by considering the dispersion of magnetic particles onto the carbonaceous material’s surface, which has a large specific surface area. Graphene is regarded as the most important carbonaceous material due to its remarkable unique properties; its applications are constrained by two key factors: its low reactivity and hydrophobicity. Oxidized forms of graphene, known as GO and RGO, are attracting attention in this direction due to the presence of surface defects and additional oxygen functional groups on their surfaces, which increase the reactivity and dispersion of the magnetic particles. In the HT process, these functional groups are valuable because they facilitate the coupling and delivery of various drugs. In addition, the increased hydrophilicity determines a good water dispersibility of the composite by forming stable colloids through coordination bonds between GO or RGO and MNPs.

The success of the HT process is contingent upon the strength of the applied magnetic field, in addition to the synthesis of Fe_3_O_4_@carbonaceous composites and the inherent properties of their components. To guarantee the biomedical, technical, and economic viability of the process, it is essential to restrict the escalation in frequency and amplitude associated with the magnetic field. In order to prevent damage to healthy tissue while maintaining effective heating of cancerous tissue, it is advised that a maximum magnetic field output of 500 kHz and a magnetic field amplitude of 10 kA/m be employed [146]. Additionally, the magnetic characteristics of the hybrids can be modified according to the synthesis methodology employed. Various conventional methods have been discussed for HT treatment in cancer patients, including wet chemical methods [65], co-precipitation [20,71,72], solvent evaporation [75], microwave hydrothermal [66], or mechanochemical procedures [136]. Furthermore, there have also been reports of synthesis methods [19,64] under more severe conditions, which are presented in Table 11 alongside the HT performances of the composites.

The effectiveness of hyperthermia in Ref. [19] is attributed to several factors, including a decrease in particle agglomeration, and an improvement in the ability to uniformly disperse particles due to chemical alteration of the Fe_3_O_4_ surface in supercritical methanol. Regarding Ref. [64], the HT performance is enhanced by the utilization of L-ascorbic acid in the synthesis route of the composite, resulting in a higher colloidal suspension than that achieved with NaBH_4_ and a higher SAR value of the composite than that of Fe_3_O_4_ pristine (11.6 W/g).

With regard to the synthesis methods employed in the production of magnetic composites utilized in HT, it is notable that the particle size of the resulting material exerts a considerable influence on the magnetic properties of the material. The phenomenon of hyperthermia is primarily attributable to three independent potential mechanisms: Brownian motion relaxation, Néel relaxation, and hysteresis loss. The relative contributions of these mechanisms are contingent upon the particle size in question. In the case of hysteresis loss, each magnetic moment aligns with the orientation of the magnetic field, whereas decreasing particle size causes the magnetic moments to decrease, resulting in a rapid change in magnetic moments (Neel relaxation) or a rotation of the particles with the changing magnetic field (Brownian relaxation) [147]. Therefore, hysteresis loss is significant for larger particles, while Brownian and Neel relaxations dominate at the nanoscale. Since the clinical environment requires small particles in a stable colloidal suspension capable of penetrating deep into tumor tissue, Neel and Brownian relaxation mechanisms should become the most dominant. As an alternative approach, measures can be implemented to enhance the stability of colloidal solutions comprising ferromagnetic particles. Based on these mechanisms, a large-scale mechanochemical synthesis route has been described in Ref. [74] to obtain GO–Fe_3_O_4_ nanocomposites with different optimized magnetic properties by adjusting the milling time. This is responsible for the size effect reduction that determines the decrease in the magnetothermal heating behavior. Alternatively, in this research study, a significant investigation has been conducted on the modification of magnetic properties by adjusting the particle size. Composites of GO-Fe_3_O_4_ with different particle sizes have been synthesized by the thermal decomposition method by varying the reaction parameters, thus modifying the saturation magnetization values [75]. For instance, the authors reveal that particles with a size of 4 and 8 nm did not exhibit the hysteresis loss mechanism, and the heating for HT is due to the Neel and Brownian mechanisms, while the heating of particles with a size of 45 nm is attributed to hysteresis loss. Due to that, the most favorable HT results were obtained for 45 nm particles, with a remarkable SAR value of 5020 W/g. The principal objective of the study was to improve the colloidal stability of ferromagnetic particles by grafting PEG onto the GO@Fe_3_O_4_ composite. Overall, the magnetic properties were observed to remain unaltered in the novel composite of GO-PEG@Fe_3_O_4_, but with remarkable colloidal stability.

### 3.3. Synergistic Effects of HT and Chemotherapy

Introducing HT as a complementary method to fight cancer can enhance the therapeutic effectiveness of the chemotherapy procedure. This is because it increases blood flow, accumulates the drug in the tumor cells, activates/improves the immune response, and enhances the tumor’s sensitivity to cytostatic drugs. The state-of-the-art combined anticancer therapy is a more effective strategy that associates the synergistic effects of high temperature from HT and the cytostatic drug action from chemotherapy. In a study evaluated by Yang S.-J. et al. [148], a powerful nanoplatform-based Fe_3_O_4_ system conjugated with cisplatin and methotrexate, as chemotherapeutic agents, employed to assess the cellular cytotoxicity associated with lung cancer treatment. The authors demonstrated the synergistic impact of HT and chemotherapy on pulmonary carcinoma and adenocarcinoma cells through in vitro tests. In vivo tests on female nude mice have shown that the tumor treated with the Fe_3_O_4_-based system experienced growth inhibition, and the neoplasm was destroyed by up to 80% when HT was induced [148]. This study suggests that HT-induced temperature increases the susceptibility of cancer cells to drug treatment by enhancing the absorption of cytostatic drugs through increased cell membrane fluidity and blood flow. In addition to these in vitro and in vivo investigations, there is some clinical information available for their validation. The synergy between HT and chemotherapy has also attracted the attention of medical clinics. For example, in localized soft tissue sarcoma, the combination of local HT and neoadjuvant chemotherapy inhibits the recurrence and metastases in patients aged 18–70, with a survival rate of 27% more than in the case of chemotherapy alone [149]. Additionally, in a clinical trial of nonmuscle-invasive bladder cancer [150], it was established that patients who underwent chemo–HT treatment exhibited a recurrence-free survival rate of 81.8%, in comparison to 64.8% for patients who received adjuvant treatment.

By integrating nanotechnology for chemo-HT, the nanoparticles can carry and release drugs into the biological environment and initiate targeted tumor heating upon an external field, leaving the surrounding tissue mainly undamaged. This innovative approach has the potential to reduce the economic impact of chemotherapy, limit its side effects, and enhance the effectiveness of the cytostatic used. Currently, various nanoparticles are used in medical studies, including porous silica, as well as organic and inorganic materials. It is worth noting that magnetic Fe_3_O_4_ is of considerable significance, and its composites with carbonaceous-based materials are currently the subject of considerable interest for chemo–HT studies, which represent a significant advance in cancer treatment [20,72,137]. CNPs (GO and RGO) allow drugs to bind to their surface through hydrophilic bonds, transport them, penetrate deep into cancer cells through endocytosis due to their amphiphilic nature, and finally release the drugs. However, Fe_3_O_4_ has the ability to target drugs to cancerous tissue using a magnetic field without heating the surrounding healthy tissue. In light of the biocompatibility studies conducted on Fe_3_O_4_–carbonaceous-based composites, it can be posited that these materials have the potential to be employed not only in a standalone HT therapy but also in conjunction with other procedures with the objective of enhancing the apoptosis or necrosis phenomenon of cancer cells. Currently, there are only a few studies focusing on composites of Fe_3_O_4_-CNPs in chemo-HT, which are still in the initial phase and require further development in the future (Table 12).

This research collectively demonstrates that the treatment of HT integrated with chemotherapy induces apoptosis in various cell lines, with a response that is significantly greater than the effect of HT or chemotherapy alone. As is expected, the procedure could be successfully applied to in vivo studies and has the potential for further advancement in clinical tests.

### 3.4. Applications in Bone Tissue Engineering

This section aims to show briefly the contribution of Fe_3_O_4_ and CNPs of the type CNTs and graphene in BTR. According to the study reported by C.Z. Liao et al., the body has the intrinsic ability to repair bone injuries, but because the process is complicated and slow, the process of adequate BTR is not adequate in the case of patients with compromised self-repair capacity [151]. Various materials for BTE, such as CNPs and Fe_3_O_4_, have emerged as an important approach for healing severe or difficult-to-repair bone injuries [152,153,154,155,156].

One of the CNPs that have attracted particular attention is CNTs [152]. This fact was a consequence of the affinity of CNTs for cells, the parameter that is significant in the BTE [154]. Moreover, the high strength and conductivity properties of CNTs allow for the reinforcement of bone scaffolds [154]. In vivo studies have demonstrated that CNTs: (i) can activate proliferation, as well as differentiation of osteoblast, and (ii) promote bone calcification and bone formation by suppressing the receptor activator of nuclear factor–κB ligands [157,158,159]. The main composites based on CNTs used as scaffolds or implants for bone tissue developed up to now were: (i) CNTs/HA [160,161]; (ii) CNTs/CS [162,163]; (iii) CNT/CS-HA [164]; (iv) CNTs/collagen [165,166]; and (v) CNTs/collagen-HA [167]. GO and RGO were the other two carbonaceous nanostructures that were reported to support osteogenic cell differentiation [155]. According to Ref. [156], composites based on GO and CS have been demonstrated to be an adequate medium for the growth and differentiation of osteoblasts, allowing an effective BTR. The HA/GO composites were reported to improve mechanical strength, viability, and, last but not least, cell proliferation, allowing for an efficient BTR [168]. The formation of new bone and spontaneous osteodifferentiation were demonstrated to be induced by the RGO/HA composites enhancing osteogenesis of the preosteoblastic cells of the type MC3T3-E1 [169]. The reconstruction of large bone defects was reported to be significant for RGO-doped poly(lactic-co-glycolic) acid (PLGA)/HA nanofiber scaffolds [170]. These composites were reported to facilitate proliferation, having a significant role in osteogenic differentiation of human MSCs [171].

Fe_3_O_4_ was another material that caught the attention of the BTE. Due to the stimulating osteoblast activity and the interaction with bone matrix proteins, these nanoparticles promote bone growth and healing after surgeries and fractures [151,172]. The study reported by A. Russo et al. in 2017 highlighted that the HA/magnetite composite with a weight ratio of 90 wt.%: 10 wt.% induces and supports the formation of bone tissue in rabbits with femoral defects [173]. To study progenitor cells of mouse bone marrow and human leukemia K562 cells, the covalent modification of Fe_3_O_4_ MNPs modified with 3-aminopropylsilane and N-di-Fmoc-L-lysine was studied in 2020 [174]. For magnetically marked cells in in vitro and in vivo experiments, the authors report a qualitative and quantitative assessment of the weight of H_2_O molecules and OH groups adsorbed on the Fe_3_O_4_ surface through FTIR spectroscopy and thermogravimetric analysis [174]. These results have highlighted a new protocol for assessing the interaction of Fe_3_O_4_ particles with cell membranes and the internalization of these in cells [174]. In 2020, Marycz K. et al. prepared nanocomposites of the type α-Fe_2_O_3_/γ-Fe_2_O_3_ by the sol–gel method for the osteoporosis treatment, when an inhibition of the osteoclast activity was demonstrated [175]. In the same year, the synthesis of Fe_2_O_3_ nanoparticles modified with polysaccharides polyglucose–sorbitol–carboxymethyl ether (PSC) was reported by Yu P. et al. for the elimination of the excess of ROS, for the inhibition of osteogenesis, and for the improvement of osteoclasts differentiation in osteoporosis [176]. The authors demonstrate that Fe_2_O_3_ nanoparticles modified with PSC are promising iron agents for the treatment of diseases with iron deficiency such as osteoporosis [176].

In light of the aforementioned characteristics, the combination of Fe_3_O_4_ and carbonaceous materials allows for the creation of a synergistic effect, whereby the magnetic and osteoblastic properties of Fe_3_O_4_ are combined with the mechanical strength and cell-supporting capacity of carbonaceous materials. Consequently, such composites may prove particularly efficacious in the clinical context, both in the regeneration of bone tissue and in the creation of resilient and adaptable bone implants. To date, only three scientific articles have concluded that the production of these nanocomposite scaffolds is effective for BTE, and they are summarized in Table 13.

## 4. Current Carbonaceous Nanomaterials for Human Health Applications

Some of the hybrid Fe_3_O_4_-CNPs obtained by the methods described above (see Table 1 and Table 2) have been tested for cytotoxicity, both before and after loading with anticancer drugs, even on human cancer cells, such as Hela (cervical carcinoma) [72,73,74,77], HepG2 (liver cell), MCF-7 (breast cancer cell) [41,56,73], HEK-293 (embryonic kidney fibroblast) [41], HBE (human bronchial epithelial) [57], BEAS 2B (lung epithelial) [57], and A549 (human lung cancer cell line) [76]. The aim of the studies performed was to evaluate the cytotoxicity of hybrid materials prepared in different proportions (Fe_3_O_4_: CNPs) to which the anti-tumor drug was bound, among those mentioned in the cited studies are doxorubicin (DOX) [62,72,76,77], daunorubicin (DNR) [58], methotrexate [73], paclitaxel (PTX) [127], hydroxycamptothecin (HCPT) [56], and 5-fluorouracil (5FU) [122]. The biocompatibility of hybrid Fe_3_O_4_–CNPs nanoparticles was found to be superior to that of pure Fe_3_O_4_ [57,61,108], where the cytotoxicity was evaluated after loading with the anti-tumor drug. According to the results obtained on the above combinations, the high efficiency in cell apoptosis observed following in vitro testing on human cancer cells encourages the next step of in vivo testing, with promising results expected.

## 5. Outlook

Despite significant progress in the utilization of carbonageous materials@Fe_3_O_4_ in drug delivery applications, a multitude of research gaps persists, thereby constraining the applicability of these systems. While there is a substantial body of in vitro research, especially for graphene derivatives@Fe_3_O_4_ nanocomposites (Table 6), it is followed by an extremely small number of in vivo experiments (Table 7 and [140,178] from Table 6). In order to gain a deeper understanding of the biocompatibility and stability of the material in question, it is necessary to conduct a series of in vivo studies utilizing relevant animal models. Additionally, the mechanisms of efficient drug release under complex human physiological conditions remain poorly understood, and there is limited information on long-term toxicity and the elimination of materials from the body.

In light of the promising synergistic approaches of HT and chemotherapy, composites comprising carbon materials@Fe_3_O_4_ are beginning to attract considerable interest within the medical community. However, there is still a notable absence of in vivo and clinical studies on these specific materials. To date, only three in vitro studies have been conducted [20,72,137], and these require further development and more detailed research to validate their efficacy. This should include an investigation of the heat distribution and its effects in complex biological conditions, with a view to optimizing the SAR value and the immunological profile of the body in response to the composite materials under investigation. It is recommended that these detailed in vitro investigations be followed by in vivo and, when appropriate, clinical studies. Furthermore, while pristine Fe_3_O_4_ has already been the subject of clinical trials [149,150], the in vivo and clinical investigations of composites comprising Fe_3_O_4_ and carbon materials have not yet reached the same level of study. This underscores the imperative for more advanced research to ascertain the potential of these composites in combinatorial HT and chemotherapy treatments.

The question thus becoming pertinent is why it is of such importance to utilize composites in medical applications such as drug delivery or the synergistic approach of HT and chemotherapy, as opposed to Fe_3_O_4_. The answer can be conceptualized by first considering the diminished cytotoxicity exhibited by Fe_3_O_4_, which is attributed to the generation of harmful ROS species (inducing oxidative stress) through the passivation of these NPs with carbonaceous materials. Additionally, the large surface area and the versatile structure of carbonaceous materials, which can encapsulate a substantial quantity of drug that subsequently needs to be released for tumor treatment, is another consequence of the preference for composite materials. Furthermore, the composites display a high capacity for functionalization with polymers (e.g., PEG and CS), proteins, or other biocompatible agents (HA), which results in good dispersibility, improved tumor targeting specificity, and increased biocompatibility, respectively. The combination of these characteristics with the specific magnetic properties of Fe_3_O_4_ renders composites based on carbonaceous materials and Fe_3_O_4_ particularly well-suited to the aforementioned applications.

**Figure 8:**
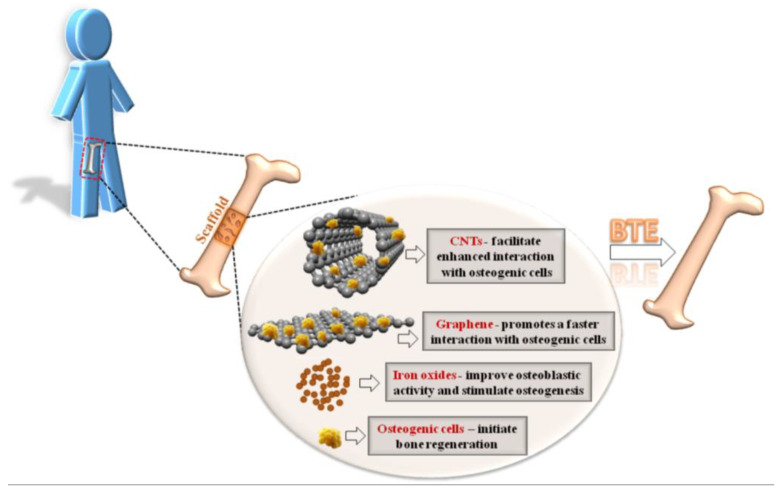
Composites of CNTs/graphene derivatives in conjunction with iron oxide nanoparticles to facilitate BTE through the stimulation of osteogenic cells.

Regarding the BTE applications, due to the findings of the three studies presented in Table 12 [23,28,177], further research into composites based on Fe_3_O_4_ and carbonaceous materials should be considered. It is, therefore, essential to establish a framework for evaluating the potential benefits and drawbacks of utilizing graphene derivatives or CNTs in future applications. It would be beneficial to consider the advantage offered by the cylindrical structure of CNTs, which allows for improved interaction with cells that facilitate BTE due to the complex and three-dimensional shape of any bone structure. However, the extended two-dimensional surface of graphene facilitates cell attachment and proliferation more effectively than CNTs, rendering it ideal for bone structures that require rapid and efficient cellular interaction, such as those subjected to the greatest mechanical pressure, namely long bones (e.g., the femur and tibia) or vertebral bones. A schematic diagram of a composite used for BTE applications, showing the advantages of CNT and graphene components, is shown in Figure 8.

The encouraging progress observed in this field indicates that nanocomposites based on Fe_3_O_4_ and carbonaceous-based nanoparticles of the type CNTs and graphene derivatives may offer significant potential in the field of BTE. It seems that the main focus is on exploring ways to enhance the stimulation of osteoblastic activity, the bioactivity of scaffolds, and their mechanical resistance, with the ultimate goal of supporting BTE. The final choice should depend on the specific type of bone defect and the mechanical and biological requirements, as well as the possibility of combination with other regenerative materials (e.g., HA). In the next period, the perspectives of the BTE research will involve the optimization of these nanocomposites regarding understanding their interactions with the biological environment, with a view to advancing clinical applications.

To make progress in biomedical applications, it is essential to develop reproducible scalable methods for the synthesis of these composites, which is a pivotal step in the transition from laboratory-scale to clinical use. Particular attention should be paid to the most preferred synthesis methods for obtaining composites of high purity. A method for obtaining iron oxides, namely co-precipitation, has already been established [148] and should be reproducible in the case of Fe_3_O_4_@carbonaceous materials composites. The co-precipitation method via flow chemistry is not only scalable, cost-effective, and straightforward but also ensures the uniform distribution of the obtained particles [179], which are in compliance with clinical safety and biocompatibility requirements. Furthermore, it is imperative to conduct comprehensive preclinical studies and investigate their potential applications. This should be done not only with the utilization of traditional chemotherapeutic agents but also with the exploration of modern therapies, such as immunotherapy. For instance, despite the potential for enhanced efficacy, the combination of drug delivery or HT with immunotherapy remains a relatively unexplored avenue of research as was highlighted in papers from 2024 [180,181]. The integration of composites with this technology has the potential to transform the field of medicine, creating multifunctional therapeutic platforms that comply with the relevant clinical regulations.

## 6. Conclusions

1. Nanohybrids based on Fe_3_O_4_ and CNPs are most commonly synthesized by co-precipitation. The primary issues addressed in this process are colloidal dispersibility, biocompatibility, drug loading, and release. The first two properties are provided by functionalization with polymers, while the last two are significantly influenced by the pH value.
2. MS and SAR are the most representative parameters that reflect the correlation between the size of the hybrid nanoparticles and the efficiency of the biomedical application. The smaller the diameter of the magnetic nanocrystallites, the higher their magnetic saturation, which consequently increases their efficiency in drug delivery.
3. In the drug loading process, the most common type of interaction is the π–π type, similar to noncovalent functionalization. This occurs between the structure of the drug molecule and the NPC component.
4. The synergic effect of hyperthermia and drugs used in chemotherapy represents a significant achievement in the field of anti-cancer therapy. The Fe_3_O_4_–CNP hybrids have been tested in vitro on human cell cultures and have been found to be biocompatible in over 90% of cases, which is a promising result for their potential use in vivo application.
5. The combination of Fe_3_O_4_-graphene proved to be the most successful combination in terms of drug delivery speed, the degree of precision, and the influence of the cell response to drug contact due to the local temperature increase.

## Figures and Tables

**Table 1:** Synthesis, nanoparticle size of Fe_3_O_4_-CNPs.

Composite	Synthesis and Interaction	Particle Size Medium Distribution	Study
GO/Fe_3_O_4_	Ball milling	20 nm	[74]
GO/Fe_3_O_4_	Thermal decomposition	4, 8, 20, 45, 250 nm	[75]
Fe_3_O_4_-RGO	Chemical co-precipitation	12.3 nm, 15.6, 13.8 nm	[61]
PEG coated Fe_3_O_4_/RGO	Microwave hydrothermal method	24–34 nm	[41]
Graphene-Fe_3_O_4_	Thermal reduction method	30 µm	[65]
GO-Fe_3_O_4_	Chemical precipitation	2–10 nm	[71]
RGO/Fe_3_O_4_	Heterocoagulation	12.6 ± 2.3 nm	[64]
PEGylated-RGO/Fe_3_O_4_	Co-precipitation	8–10 nm, tested at 265 kHz for 35 min	[72]
Fe_3_O_4_-GO	Precipitation	1325 nm	[57]
Fe_3_O_4_-graphene	Supercritical method	-	[19]
Fe_3_O_4_-GO	Chemical precipitation	15–30 nm	[20]
Fe_3_O_4_-GO (modified with CS and sodium alginate)	LbL self-assembly technique	0.5 μm mGO-CS/SA	[76]
PEG-Fe_3_O_4_-GO	Chemical co-precipitation	20 nm Fe_3_O_4_	[73]
GO/Fe_3_O_4_	Chemical precipitation	260 nm	[77]
rGO/Fe_3_O_4_ CS overlaid	Solvothermal method	<35 nm in diameter for Fe_3_O_4_ NPs	[63]
GO/Fe_3_O_4_	Co-precipitation	239.8 ± 0.08 nm	[60]
PEG/graphene/Fe_3_O_4_	Solvothermalmethod	Unspecified	[62]
GO/Fe_3_O_4_/Au	Co-precipitation	17.36 nm	[59]
Fe_3_O_4_/GO glucosamine (GA) conjugated, nanohybrids	Co-precipitation	16.12 nm	[58]
GO/Fe_3_O_4_	Co-precipitation	-	[56]
FA-Fe_3_O_4_-MWCNTs	Chemical precipitation	<10 nm in diameter attached to the MWCNTs surface	[78]
Fe_3_O_4_-MWCNTs	Co-precipitation	Not specified	[79]
CNT/Fe_3_O_4_ (H4,fCNT/ION Fe^2+^/Fe^3+^) = 1:4	Co-precipitation	6 to 30 nm	[23]
CNT-COOH/Fe_3_O_4_	-	Unspecified	[24]

**Table 2:** Synthesis of Fe_3_O_4_–CNP composites and magnetic characteristics.

Composite	Synthesis and Interaction	Particle Size	SAR	M_S_	Study
GO/Fe_3_O_4_	Ball milling	20 nm	70 W/g	-	[74]
GO/Fe_3_O_4_	Thermal decomposition	4, 8, 20, 45, 250 nm	5020 W/g (45 nm)	-	[75]
PEG-coated Fe_3_O_4_/RGO	Microwave hydrothermal	24–34 nm	58.33 W/g	-	[41]
Graphene-Fe_3_O_4_	Thermal reduction	30 µm	6.45 W/g	Mr = 6 emu/g Ms = 20 emu/g	[65]
GO-Fe_3_O_4_	Precipitation	2–10 nm Fe_3_O_4_	-	Ms = 47.56 emu/g Mr = 0.173 emu/g	[71]
RGO/Fe_3_O_4_	Heterocoagulation	12.6 ± 2.3 nm	20.3 W/g	-	[64]
PEGylated-RGO/Fe_3_O_4_	Co-precipitation	8–10 nm	5000 W/g	-	[72]
Fe_3_O_4_-graphene	Supercritical method	-	-	Ms GO = 31.1 emu/g and Ms RGO = 78.8 emu/g	[19]
Fe_3_O_4_-GO	Precipitation	15–30 nm Fe_3_O_4_	-	Ms(1:1) = 47.56 emu/g Mr = 3.808 emu/g	[20]
Fe_3_O_4_-GO	LbL self-assembly technique	0.5 μm	-	Ms = 38 emu/g	[76]
Fe_3_O_4_@GO-PEG	Co-precipitation	20 nm Fe_3_O_4_	-	57.6 emu/g for GO-Fe_3_O_4_; 51.8 emu/g for GO-PEG@Fe_3_O_4_	[73]
RGO-CS/Fe_3_O_4_	Solvothermal method	<35 nm	-	Ms = 5.27 emu/g Mr = 1.23 emu/g	[63]
GO/Fe_3_O_4_	Co-precipitation	239.8 ± 0.08 nm	-	Ms = 58.42 emu/g	[60]
PEG-graphene@ Fe_3_O_4_	Solvothermal	Unspecified	-	Ms = 31.052 emu/g for MG-NH2- vs. Ms = 4.595 emu/g for MG-NH2-PEG	[62]
GO/Fe_3_O_4_/Au	Co-precipitation	17.36 nm	-	Ms = 29.2 (emu/g)	[59]
Fe_3_O_4_/GO–GA conjugated, nanohybrids	Co-precipitation	16.12 nm	-	Ms = 26.017 emu/g)	[58]
FA-Fe_3_O_4_-MWCNTs	Precipitation	<10 nm	-	Ms = 68.3 emu/g, Mr = 0 emu/g	[78]
CNT/Fe_3_O_4_ (H4, CNT/ION Fe^2+^/Fe^3+^) = 1:4	Co-precipitation	6 to 30 nm	-	43 emu/g	[23]

**Table 3:** Studies implying biocompatibility of Fe_3_O_4_@carbonaceous based materials.

Composites	Cells	Incubation Period	Cell Viability	Apoptotic Rate	Ref.
Fe_3_O_4_@GO (c = 10 µg/mL)	HBE BEAS-2B	12 h	80% 60%	- -	[57]
Fe_3_O_4_@GO (c = 200 µg/mL)	HBE BEAS-2B	12 h	60% 40%	- -	[57]
Fe_3_O_4_@GO	Mesenchymal stem cells	12 h 48 h	80–90% >100%	~15% (c = 10 µg/mL)	[108]
Fe_3_O_4_@RGO	HepG2 cells	-	90–100%	Minimum	[61]

**Table 4:** Preliminary studies of graphene-based composites with Fe_3_O_4_ used in drug delivery.

Graphene-Based Composites	Polymers	Drug Concentration	Drug Loading Efficiency	Ref.
Fe_3_O_4_@graphene quantum dots	Chitosan	2 mg/mL 5-FU	90%—8 h 70–84%—48 h	[122]
Fe_3_O_4_@izolated graphene layers	PVP and PVA	0.02 mg/mL DOX 0.02 mg/mL PTXL	87% 91%	[123]

**Table 5:** Studies of graphene derivatives-based composites with Fe_3_O_4_ used in drug delivery.

Graphene Derivatives-Based Composites	Polymer Integration	Drug Concentration	Drug Loading Efficiency	Ref.
GO@Fe_3_O_4_	PEG	1 µg/mL DOX	95%	[124]
		0.2 mg/mL DOX	40%	[125]
		1.6 mg/mL DOX	220%	[125]
GO@Fe_3_O_4_ RGO@Fe_3_O_4_	CS	0.2 mg/mL DOX	137%	[76]
		0.5 mg/mL DOX	95%	[63]
GO@Fe_3_O_4_	GA	0.05–1 mg/mL Daunorubicin	93%	[58]

**Table 6:** Drug release efficiency of graphene-based composites with Fe_3_O_4_ dependent on the pH medium.

Graphene-Based Composites	Drug Action	Drug Concentration Drug Loading Efficiency		Ref.
		Basic/Neutral Medium	Acidic Medium	
GO@Fe_3_O_4_	DOX	66.1%	97.3%	[130]
RGO-CS@Fe_3_O_4_	DOX	10%	96.6%	[63]
Graphene quantum dots -CS@Fe_3_O_4_	5-FU	69.3%	84%	[122]
GO@Fe_3_O_4_	5-FU	21%	72%	[10]
GO@Fe_3_O_4_	MTX	78%	92%	[129]

**Table 7:** Cellular uptake and cytotoxicity of drug-conjugated nanocarriers.

Composites	Drug Penetration	Malignant Cell Internalization	Incubation Period	Composite Biocompatibility	Tumor Cytotoxicity	Ref.
RGO@Fe_3_O_4_	0.5 mp/mL 5-FU	HepG2	3 h	98%	-	[127]
Graphene@ Fe_3_O_4_	50 mg/mL DOX 50 mg/mL PTXL	HeLa	8 h	>80%	54% 44%	[123]
RGO-CS@ Fe_3_O_4_	0.5 mg/mL DOX	A549 MCF-7	24 h	High	91.4% 92.7%	[63]
GO-PEG@ Fe_3_O_4_	32 µg/mL DOX	MCF-7	48 h	95–100%	95%	[130]
GO-PEG@ Fe_3_O_4_	9 µmol/L DOX	4T1	24 h	82%	YES	[125]

**Table 8:** Studies of CNT-based composites with iron oxides used in drug delivery.

Composites	Drug	Malignant Cells	Incubation Time	Cytotoxicity	Drug Release	Ref.
Fe_3_O_4_@SWCNT (+poly(lactide) eco-methoxy PEG micelle)	50 µg/mL Docetaxel	MCF7	72 h	64%	43.7% (after 340 h)	[22]
γ-Fe_2_O_3_@MWCNT (+PEG@poly citric acid)	100 µg/mL Cisplatin	L929	72 h	95%	-	[134]

**Table 9:** Schematic representation of the in vivo study comprising Fe_3_O_4_ and MWCNTs.

Study	Composite	Drug	In Vivo Study	Composite@drug Absorption in Lymph		Ref.
				Without Magnetic Field	With Magnetic Field	
Lymph node metastasis	Fe_3_O_4_@ MWCNT	Gemcitabine	Sprague Dawley rat	6 µg/g	9 µg/g	[79]

**Table 10:** Schematic representation of the in vitro study comprising Fe_3_O_4_ and MWCNTs.

Study	Composite	Cell	Drug	Drug Loading Efficiency	Drug Release Efficiency		Ref.
					Basic pH	Acidic pH	
Malignant cerebral tumor	Fe_3_O_4_@MWCNT (+folic acid)	U87	DOX	96%	14%	71%	[78]

**Table 11:** HT performances in composites synthesized in stringent conditions.

Composites	Synthesis Conditions	Magnetization	SAR	Ref.
RGO@Fe_3_O_4_	Atmosphere of methanol (p = 300 atm, t = 400 °C)	1.9 higher than in conventional composites	-	[19]
RGO@Fe_3_O_4_ (1/5)	NaBH_4_ L-ascorbic acid (basic solution)	- -	12.7 W/g 20.3 W/g	[64]

**Table 12:** Chemo–HT preliminary studies comprising carbonaceous materials and Fe_3_O_4_.

Drug Transporter	Cancer Cells	Chemotherapeutic Agent	Drug Release	Cytotoxicity		Ref.
				Without Magnetic Field	With Magnetic Field	
GO@Fe_3_O_4_	-	DOX	30% (pH = 7.4) (after 20 h)	-	-	[20]
RGO@Fe_3_O_4_	HeLa	DOX	-	50%	90%	[72]
GO@Fe_3_O_4_	MCF-7	Steroidal diamine dimer	-	Almost all cells died		[137]

**Table 13:** Carbonaceous-based materials and Fe_3_O_4_ as potential nanocomposite scaffolds for BTE.

Composite	Active Medium	Composite Characteristics for BTE	Ref.
GO@Fe_3_O_4_	Mesenchyme cells from the bone marrow of a rat	Stimulates the process of regeneration and the formation of new bone tissue	[28]
GO@Fe_3_O_4_	Polymeric scaffold containing hydroxyapatite and CS	Enhances the mechanical resistance a 23-fold increase in compressive strength was observed in comparison with the control sample.	[177]
CNT@Fe_3_O_4_	Porous poly(ε-caprolactone) scaffold	Influences the cell adhesion Stimulates the metabolic activity	[23]

## Data Availability

Data sharing is not applicable. No new data were created or analyzed in this study.

## Abbreviation

5-FU	Fluorouracil
AFM	Atomic force microscopy
BTE	Bone tissue engineering
BTR	Bone tissue regeneration
CNPs	Carbonaceous nanoparticles
CNTs	carbon nanotubes
CS	Chitosan
CVD	Chemical vapor deposition
DEG	Diethylene glycol
DI	Deionized water
DNA	Deoxyribonucleic acid
DNR	Daunorubicin
DOX	Doxorubicin hydrochloride
EDC	1-(3-dimethylaminopropyl)-3-ethylcarbodiimide
EG	Ethylene glycol
EMA	European Medicines Agency
EPR	Electron Paramagnetic Resonance Spectroscopy
fCNTs	Functionalized carbon nanotubes
FDA	US Food and Drug Administration
Fe_3_O_4_	Iron oxide
FeCl_2_	Iron(II) chloride/ferrous chloride
FeCl_3_	Iron(III) chloride/ferric chloride
FTIR	Fourier transform infrared
GA	Glucosamine
G–NH_2_	Amine-modified graphene
GO	Graphene oxide
H_2_O	Water
HA	Hydroxyapatite
Hc	Coercivity
Hsp70	Protein 70
HT	Hyperthermia
LbL	Layer-by-layer
MNPs	Magnetic nanoparticles
MRI	magnetic resonance imaging
MSCs	Mesenchymal stem cells
M_S_	Magnetic saturation
MTX	Methotrexate
MWCNTs	Multi-walled carbon nanotubes
NH_4_OH	Ammonium hydroxide
NHS	N-hydroxysuccinimide
NPs	Nanoparticles
PCL	Poly(ε-caprolactone)
PEG	Polyethylene glycol
PLGA	Poly(lactic-co-glycolic)
PSC	Polysaccharides polyglucose–sorbitol–carboxymethyl ether
PTXL	Paclitaxel
RGO	Reduced graphene oxide
ROS	Reactive oxygen species
SAR	Specific absorption rate
SEM	Scanning electron microscopy
SWCNTs	Single-walled carbon nanotubes
TEM	Transmission electron microscopy
VSM	Vibrating sample magnetometer
XRD	X-ray diffraction
XPS	X-ray photoelectron spectroscopy

## References

[1] Yin P.T., Shah S., Chhowalla M., Lee K.-B. (2015). Design, Synthesis, and Characterization of Graphene–Nanoparticle Hybrid Materials for Bioapplications. Chem. Rev..

[2] Iijima S. (1991). Helical Microtubules of Graphitic Carbon. Nature.

[3] Kostarelos K., Bianco A., Prato M. (2009). Promises, Facts, and Challenges for Carbon Nanotubes in Imaging and Therapeutics. Nat. Nanotechnol..

[4] Liu Z., Tabakman S.M., Chen Z., Dai H. (2009). Preparation of Carbon Nanotube Bioconjugates for Biomedical Applications. Nat. Protoc..

[5] Geim A.K., Novoselov K.S. (2007). The Rise of Graphene. Nat. Mater..

[6] Novoselov K.S., Geim A.K., Morozov S.V., Jiang D., Zhang Y., Dubonos S.V., Grigorieva I.V., Firsov A.A. (2004). Electric Field Effect in Atomically Thin Carbon Films. Science.

[7] Lu C.H., Zhu C.L., Li J., Liu J.J., Chen X., Yang H.H. (2010). Using Graphene to Protect DNA from Cleavage During Cellular Delivery. Chem. Commun..

[8] Depana D., Shah J., Misra R.D.K. (2011). Controlled Release of Drug from Folate-Decorated and Graphene-Mediated Drug Delivery System: Synthesis, Loading Efficiency, and Drug Release Response. Mat. Sci. Eng. C.

[9] Wu H.X., Liu G., Wang X., Zhang J.M., Chen Y., Shi J.L., Yang H., Hu H., Yang S.P. (2011). Multifunctional Mesoporous Silica Nanoparticles as a Theranostic Platform for Imaging-Guided Cancer Therapy. Acta Biomater..

[10] Wang G., Chen G., Wei Z., Dong X., Qi M. (2013). Multifunctional Fe_3_O_4_/graphene oxide nanocomposites for magnetic resonance imaging and drug delivery. Mater. Chem. Phys..

[11] Faraj A.A., Shaik A.P., Shaik A.S. (2015). Magnetic Single-Walled Carbon Nanotubes as Efficient Drug Delivery Nanocarriers in a Breast Cancer Murine Model: Noninvasive Monitoring Using Diffusion-Weighted Magnetic Resonance Imaging as a Sensitive Imaging Biomarker. Int. J. Nanomedicine.

[12] Cunha C., Panseri S., Iannazzo D., Piperno A., Pistone A., Fazio M., Russo A., Marcacci M., Galvagno S. (2012). Hybrid composites made of multiwalled carbon nanotubes functionalized with Fe_3_O_4_ nanoparticles for tissue engineering applications. Nanotechnology.

[13] Bianco A., Kostarelos K., Prato M. (2011). Making Carbon Nanotubes Biocompatible and Biodegradable. Chem. Commun..

[14] Moradi S., Akhavan O., Tayyebi A., Rahighi R., Mohammadzadeh M., Rad H.S. (2015). Magnetite/dextran-functionalized graphene oxide nanosheets for in vivo positive contrast magnetic resonance imaging. RSC Adv..

[15] Tharmarajah L. (2018). Complementary and alternative therapies for breast cancer worldwide. Lett. Health. Biol. Sci..

[16] Ji S.R., Liu C., Zhang B., Yang F., Xu J., Long J., Jin C., Fu D.L., Ni Q.X., Yu X.J. (2010). Carbon nanotubes in cancer diagnosis and therapy. BBA-Rev. Cancer.

[17] Moon H.K., Lee S.H., Choi H.C. (2009). In vivo near-infrared mediated tumor destruction by photothermal effect of carbon nanotubes. ACS Nano..

[18] Yaqoob Z., Batool S.A., Basit M.A., Konain K., Malik R.A., Rehman S.U., Hussain S.W., Alrobei H., Rehman M.A.U. (2024). Hydrothermal synthesis of carbon dots incorporated in magnetite iron oxide nanoparticles for potential targeted brain cancer therapy: In-Vitro study. Mater. Chem. Phys..

[19] Tayyebi A., Moradi S., Azizi F., Outokesh M., Shadanfar K., Mousavi S.S. (2017). Fabrication of new magnetite-graphene nanocomposite and comparison of its laser-hyperthermia properties with conventionally prepared magnetite-graphene hybrid. Mat. Sci. Eng. C Mater..

[20] Ren M.-X., Wang Y.-Q., Lei B.-Y., Yang X.-X., Hou Y.-L., Meng W.-J., Zhao D.L. (2021). Magnetite nanoparticles anchored on graphene oxide loaded with doxorubicin hydrochloride for magnetic hyperthermia therapy. Ceram. Int..

[21] Shimizu K., Ito A., Honda H. (2007). Mag-seeding of rat bone marrow stromal cells into porous hydroxyapatite scaffolds for bone tissue engineering. J. Biosci. Bioeng..

[22] Gungunes C.D., Seker S., Elcin A.E., Elcin Y.M. (2017). A comparative study on the in vitro cytotoxic responses of two mammalian cell types to fullerenes, carbon nanotubes and iron oxide nanoparticles. Drug Chem. Toxicol..

[23] Świętek M., Brož A., Tarasiuk J., Wroński S., Tokarz W., Kozieł A., Błażewicz M., Bačáková L. (2019). Carbon nanotube/iron oxide hybrid particles and their PCL-based 3D composites for potential bone regeneration. Mater. Sci. Eng. C Mater..

[24] Rezaei A., Morsali A., Bozorgmehr M.R., Nasrabadi M. (2021). Quantum chemical analysis of 5-aminolevulinic acid anticancer drug delivery systems: Carbon nanotube, –COOH functionalized carbon nanotube and iron oxide nanoparticle. J. Mol. Liq..

[25] Chen M.-L., He Y.-J., Chen X.-W., Wang J.-H. (2012). Quantum dots conjugated with Fe_3_O_4_-filled carbon nanotubes for cancer-targeted imaging and magnetically guided drug delivery. Langmuir.

[26] Pistone A., Iannazzo D., Panseri S., Montesi M., Tampieri A., Galvagno S. (2014). Hydroxyapatite-magnetite-MWCNT nanocomposite as a biocompatible multifunctional drug delivery system for bone tissue engineering. Nanotechnology.

[27] Fan X., Jiao G., Gao L., Jin P., Li X. (2013). The preparation and drug delivery of a graphene-carbon nanotube-Fe_3_O_4_ nanoparticle hybrid. J. Mater. Chem. B.

[28] Niu Z., Murakonda G.K., Jarubula R., Dai M. (2021). Fabrication of graphene oxide-Fe_3_O_4_ nanocomposites for application in bone regeneration and treatment of leukemia. J. Drug Deliv. Sci. Tec..

[29] Lee X.J., Lim H.N., Gowthaman N.S.K., Rahman M.B.A., Abdullah C.A.C., Muthoosamy K. (2020). In-situ surface functionalization of superparamagnetic reduced graphene oxide—Fe_3_O_4_ nanocomposite via Ganoderma lucidum extract for targeted cancer therapy application. Appl. Surf. Sci..

[30] Su X., Chan C., Shi J., Tsang M.K., Pan Y., Cheng C., Gerile O., Yang M. (2017). A graphene quantum dot@Fe_3_O_4_@SiO_2_ based nanoprobe for drug delivery sensing and dual-modal fluorescence and MRI imaging in cancer cells. Biosens. Bioelectron..

[31] Radzi M.R.M., Johari N.A., Zawawi W.F.A.W.M., Zawawi N.A., Latiff N.A., Malek N.A.N.N., Wahab A.A., Salim M.I., Jemon K.E. (2022). In vivo evaluation of oxidized multiwalled-carbon nanotubes-mediated hyperthermia treatment for breast cancer. Biomater. Adv..

[32] Jia G., Wang H., Yan L., Wang X., Pei R., Yan T., Zhao Y., Guo X. (2005). Cytotoxicity of carbon nanomaterials: Single-walled nanotube, multi-wall nanotube, and fullerene. Environ. Sci. Technol..

[33] Ding L., Stilwell J., Zhang T., Elboudwarej O., Jiang H., Selegue J.P., Cooke P.A., Gray J.W., Chen F.F. (2005). Molecular characterization of the cytotoxic mechanism of multiwall carbon nanotubes and nano-onions on human skin fibroblast. Nano Lett..

[34] Panahi F.H., Peighambardoust S.J., Davaran S., Salehi R. (2017). Development and characterization of PLA-mPEG copolymer containing iron nanoparticle-coated carbon nanotubes for controlled delivery of Docetaxel. Polymer.

[35] Xiao D., Dramou P., He H., Pham-Huy L.A., Li H., Yao Y., Pham-Huy C. (2012). Magnetic carbon nanotubes: Synthesis by a simple solvothermal process and application in magnetic targeted drug delivery system. J. Nanopart. Res..

[36] Cao S.W., Zhu Y.J., Chang J. (2008). Fe_3_O_4_ polyhedral nanoparticles with a high magnetization synthesized in mixed solvent ethylene glycol–water system. New J. Chem..

[37] Shen W., Chen X., Shi Y., Shi M., Chen H. (2012). Synthesis of monodisperse and single-crystal Fe_3_O_4_ hollow spheres by a solvothermal approach. Mater. Chem. Phys..

[38] Xuan S., Wang Y.-X.J., Yu J.C., Cham-Fai L.K. (2009). Tuning the grain size and particle size of superparamagnetic Fe_3_O_4_ microparticles. Chem. Mater..

[39] Cherukuri P., Bachilo S.M., Litovsky S.H., Weisman R.B. (2004). Near-infrared fluorescence microscopy of single-walled carbon nanotube in phagocytic cells. J. Am. Chem. Soc..

[40] Heller D.A., Baik S., Eurell T.E., Strano M.S. (2005). Single-walled carbon nanotube spectroscopy in live cells: Towards long-term labels and optical sensors. Adv. Mater..

[41] Vittorio O., Duce S.L., Pietrabissa A., Cuschieri A. (2011). Multiwall carbon nanotubes as MRI contrast agents for tracking stem cells. Nanotechnology.

[42] Singh R., Pantarotto D., Lacerda L., Pastorin G., Klumpp C., Prato M., Bianco A., Kostarelos K. (2006). Tissue biodistribution and blood clearance rates of intravenously administered carbon nanotube radiotracers. Proc. Natl. Acad. Sci. USA.

[43] Singh R., Pantarotto D., McCarthy D., Chaloin O., Hoebeke J., Partidos C.D., Briand J.P., Prato M., Bianco A., Kostarelos K. (2005). Binding and condensation of plasmid DNA onto functionalized carbon nanotubes: Toward the construction of nanotube-based gene delivery vectors. J. Am. Chem. Soc..

[44] Chen R.J., Choi H.C., Bangsaruntip S., Yenilmez E., Tang X.W., Wang Q., Chang Y.L., Dai H.J. (2004). An investigation of the mechanisms of electronic sensing of protein adsorption on carbon nanotube devices. J. Am. Chem. Soc..

[45] Wu W., Wieckowski S., Pastorin G., Benincasa M., Klumpp C., Briand J.-P., Gennaro R., Prato M., Bianco A. (2005). Targeted delivery of amphotericin B to cells by using functionalized carbon nanotubes. Angew. Chem. Int. Ed..

[46] Pastorin G., Wu W., Wieckowski S., Kostarelos K., Briand J.-P., Prato M., Bianco A. (2006). Double functionalisation of carbon nanotubes for multimodal drug delivery. Chem. Commun..

[47] Kostarelos K., Lacerda L., Pastorin G., Wu W., Wieckowski S., Luangsivilay J., Godefroy S., Pantarotto D., Briand J.-P., Muller S. (2007). Cellular uptake of functionalized carbon nanotubes is independent of functional group and cell type. Nat. Nanotechnol..

[48] Al-Jamal K.T., Nerl H., Müller K.H., Ali-Boucetta H., Li S., Hanes P.D., Jinschek J.R., Prato M., Bianco A., Kostarelos K. (2011). Cellular uptake mechanisms of functionalised multi-walled carbon nanotubes by 3D electron tomography imaging. Nanoscale.

[49] Dumortier H., Lacotte S., Pastorin G., Marega R., Wu W., Bonifazi D., Briand J.-P., Prato M., Muller S., Bianco A. (2006). Functionalized Carbon Nanotubes Are Non-Cytotoxic andPreserve the Functionality of Primary Immune Cells. Nano Lett..

[50] Muller J., Delos M., Panin N., Rabolli V., Huaux F., Lison D. (2009). Absence of carcinogenic response to multi-wall carbon nanotubes in a 2-year bioassay in the peritoneal cavity of the rat. Toxicol. Sci..

[51] Sayes C.M., Liang F., Hudson J.L., Mendez J., Guo W., Beach J.M., Moore V.C., Doyle C.D., West J.L., Billups W.E. (2006). Functionalization density dependence of single-walled carbon nanotubes cytotoxicity in vitro. Toxicol. Lett..

[52] Stopin A., Pineux F., Marega R., Bonifazi D. (2015). Magnetically Active Carbon Nanotubes at Work. Chem. Eur. J..

[53] Peng E., Choo E.S.G., Chandrasekharan P., Yang C.-T., Ding J., Chuang K.-H., Xue J.M. (2012). Synthesis of manganeseferrite/graphene oxide nanocomposites for biomedicalapplications. Small.

[54] Li D., Muller M.B., Gilje S., Kaner R.B., Wallace G.G. (2008). Processable aqueous dispersions of graphene nanosheets. Nat. Nanotechnol..

[55] Tien H.W., Huang Y.L., Yang S.Y., Hsiao S.T., Liao W.H., Li M.H., Wang Y.S., Wang J.Y., Ma C.C.M. (2012). Preparation of transparent, conductive films by graphene nanosheet deposition on hydrophilic or hydrophobic surfaces through control of the pH value. J. Mater. Chem..

[56] Jedrzejczak-Silicka M., Szymańska K., Mijowska E., Rakoczy R. (2024). The Influence of Graphene Oxide-Fe_3_O_4_ Differently Conjugated with 10-Hydroxycamptothecin and a Rotating Magnetic Field on Adenocarcinoma Cells. Int. J. Mol. Sci..

[57] Zhang Y., Yang Z., Fan Y., Chen M., Zhao M., Bo Dai B., Zheng L., Zhang D. (2022). Cytotoxicity Effect of Iron Oxide (Fe_3_O_4_)/Graphene Oxide (GO) Nanosheets in cultured HBE Cells. Front. Chem..

[58] Mousavi M., Salehi Z., Rezvanpour A., Mosayebi M., Farahani M., Tokmedash M.A., Shokrgozar M.A. (2023). Glucosamine conjugated iron oxide and graphene oxide nanohybrid for smart drug delivery. Can. J. Chem. Eng..

[59] Saqezi A.S., Kermanian M., Ramazani A., Sadighian S. (2022). Synthesis of Graphene Oxide/Iron Oxide/Au Nanocomposite for Quercetin Delivery. J. Inorg. Organomet. P..

[60] Wang L.-H., Sui L., Zhao P.-H., Ma H.-D., Liu J.-Y., Wei Z., Zhan Z.-J., Wang Y.-L. (2020). A composite of graphene oxide and iron oxide nanoparticles for targeted drug delivery of temozolomide. Pharmazie.

[61] Ahamed M., Akhtar M.J., Khan M.A.M. (2020). Investigation of cytotoxicity, apoptosis, and oxidative stress response of Fe_3_O_4_-RGO nanocomposites in human liver HepG2 cells. Materials.

[62] Farani M.R., Khadiv-Parsi P., Riazi G.H., Ardestani M.S., Rad H.S. (2020). PEGylation of graphene/iron oxide nanocomposite: Assessment of release of doxorubicin, magnetically targeted drug delivery, and photothermal therapy. Appl. Nanosci..

[63] Karthika V., AlSalhi M.S., Devanesan S., Gopinath K., Arumugam A., Govindarajan M. (2020). Chitosan overlaid Fe_3_O_4_/rGO nanocomposite for targeted drug delivery, imaging, and biomedical applications. Sci. Rep..

[64] Illés E., Tombácz E., Hegedűs Z., Szabó T. (2020). Tunable magnetic hyperthermia properties of pristine and mildly reduced graphene oxide/magnetite nanocomposite dispersions. Nanomaterials.

[65] Dar M.S., Akram K.B., Sohail A., Arif F., Zabihi F., Yang S., Munir S., Zhu M., Abid M., Nauman M. (2021). Heat induction in two-dimensional graphene–Fe_3_O_4_ nanohybrids for magnetic hyperthermia applications with artificial neural network modeling. RSC Adv..

[66] Alkhayal A., Fathima A., Alhasan A.H., Alsharaeh E.H. (2021). PEG-coated Fe_3_O_4_/RGO nano-cube-like structures for cancer therapy via magnetic hyperthermia. Nanomaterials.

[67] Krishna R., Fernandes D.M., Venkataramana E., Dias C., Ventura J., Freire C., Titus E. (2015). Improved reduction of graphene oxide. Mater. Today Proc..

[68] Abdolhosseinzadeh S., Asgharzadeh H., Kim H.S. (2015). Fast and fully-scalable synthesis of reduced graphene oxide. Sci. Rep..

[69] Zhu C., Guo S., Fang Y., Dong S. (2010). Reducing sugar: New functional molecules for the green synthesis of graphene nanosheets. ACS Nano.

[70] Bansal K., Singh J., Dhaliwal A.S. (2022). Synthesis and characterization of graphene oxide and its reduction with different reducing agents. IOP Conf. Ser. Mater. Sci. Eng..

[71] Bai L.-Z., Zhao D.-L., Xu Y., Zhang J.-M., Gao Y.-L., Zhao L.-Y., Tang J.-T. (2012). Inductive heating property of graphene oxide–Fe_3_O_4_ nanoparticles hybrid in an AC magnetic field for targeted drug delivery. Mater. Lett..

[72] Gupta J., Prakash A., Jaiswal M.K., Agarwal A., Bahadur D. (2018). Superparamagnetic iron oxide-reduced graphene oxide nanohybrid: A vehicle for targeted drug delivery and hyperthermia treatment of cancer. J. Magn. Magn. Mater..

[73] Abdollahi Z., Taheri-Kafrani A., Bahrani S.A., Kajani A.A. (2019). PEGylated graphene oxide/superparamagnetic nanocomposite as a high-efficiency loading nanocarrier for controlled delivery of methotrexate. J. Biotechnol..

[74] Narayanaswamy V., Obaidat I.M., Kamzin A.S., Latiyan S., Jain S., Kumar H., Srivastava C., Alaabed S., Issa B. (2019). Synthesis of graphene oxide-Fe_3_O_4_ based nanocomposites using the mechanochemical method and in vitro magnetic hyperthermia. Int. J. Mol. Sci..

[75] Sugumaran P.J., Liu X.-L., Herng T.S., Peng E., Ding J. (2019). GO-functionalized large magnetic iron oxide nanoparticles with enhanced colloidal stability and hyperthermia performance. ACS Appl. Mater. Inter..

[76] Xie M., Feng Zhang F., Peng H., Zhang Y., Li Y., Xu Y., Xie J. (2019). Layer-by-layer modification of magnetic graphene oxide by chitosan and sodium alginate with enhanced dispersibility for targeted drug delivery and photothermal therapy. Colloid Surface B.

[77] Gonzalez-Rodriguez R., Campbell E., Naumov A. (2019). Multifunctional graphene oxide/iron oxide nanoparticles for magnetic targeted drug delivery, dual magnetic resonance/fluorescence imaging, and cancer sensing. PLoS ONE.

[78] Lu Y.-J., Wei K.-C., Ma C.-C.M., Yang S.Y., Chen J.P. (2012). Dual targeted delivery of doxorubicin to cancer cells using folate-conjugated magnetic multi-walled carbon nanotubes. Colloid. Surface B.

[79] Yang D., Yang F., Hu J., Long J., Wang C., Fu D., Nib Q. (2009). Hydrophilic multi-walled carbon nanotubes decorated with magnetite nanoparticles as lymphatic targeted drug delivery vehicles. Chem. Commun..

[80] Lu D., Wu X., Wang W., Ma C., Pei B., Wu S. (2021). Synthesis and application of iron oxide nanoparticles in bone tissue repair. J. Nanomater..

[81] Pucci C., Degl’Innocent A., Gümüş M.B., Ciofani G. (2022). Superparamagnetic iron oxide nanoparticles for magnetic hyperthermia: Recent advancements, molecular effects, and future directions in the omics era. Biomater. Sci..

[82] Vergés M.A., Costo R., Roca A.G., Marco J.F., Goya G.F., Serna C.J., Morales M.P. (2008). Uniform and water stable magnetite nanoparticles with diameters around the monodomain–multidomain limit. J. Phys. D Appl. Phys..

[83] Périgo E.A., Hemery G., Sandre O., Ortega D., Garaio E., Plazaola F., Teran F. (2015). Fundamentals and advances in magnetic hyperthermia. J. Appl. Phys. Rev..

[84] Dennis C.L., Ivkov R. (2013). Physics of heat generation using magnetic nanoparticles for hyperthermia. Int. J. Hyperthermia.

[85] Hervault A., Thanh N.T.K. (2014). Magnetic nanoparticle-based therapeutic agents for thermo-chemotherapy treatment of cancer. Nanoscale.

[86] Bassetto M., Ajoy D., Poulhes F., Obringer C., Walter A., Messadeq N., Sadeghi A., Puranen J., Ruponen M., Kettunen M. (2021). Magnetically Assisted Drug Delivery of Topical Eye Drops Maintains Retinal Function In Vivo in Mice. Pharmaceutics.

[87] Szczęch M., Orsi D., Łopuszyńska N., Cristofolini L., Jasiński K., Węglarz W.P., Albertini F., Kereïche S., Szczepanowicz K. (2020). Magnetically responsive polycaprolactone nanocarriers for application in the biomedical field: Magnetic hyperthermia, magnetic resonance imaging, and magnetic drug delivery. RSC Adv..

[88] Puiu R.A., Balaure P.C., Constantinescu E., Grumezescu A.M., Andronescu E., Oprea O.C., Vasile B.S., Grumezescu V., Negut I., Nica I.C. (2021). Anti-Cancer Nanopowders and MAPLE-Fabricated Thin Films Based on SPIONs Surface Modified with Paclitaxel Loaded β-Cyclodextrin. Pharmaceutics.

[89] Ovejero J.G., Cabrera D., Carrey J., Valdivielso T., Salas G., Teran F. (2016). Effects of inter- and intra-aggregate magnetic dipolar interactions on the magnetic heating efficiency of iron oxide nanoparticles. J. Phys. Chem. Chem. Phys..

[90] Kermanian M., Sadighian S., Ramazani A., Naghibi M., Khoshkam M., Ghezelbash P. (2021). Inulin-Coated Iron Oxide Nanoparticles: A Theranostic Platform forContrast-Enhanced MR Imaging of Acute Hepatic Failure. ACS Biomater. Sci. Eng..

[91] Dulińska-Litewka J., Lazarczyk A., Halubiec P., Szafranski O., Karnas K., Karewicz A. (2019). Superparamagnetic iron oxide nanoparticles—Current and prospective medical applications. Materials.

[92] Vangijzegem T., Lecomte V., Ternad I., Van Leuven L., Muller R.N., Stanicki D., Laurent S. (2023). Superparamagnetic iron oxide nanoparticles (SPION): From fundamentals to state-of-the-art innovative applications for cancer therapy. Pharmaceutics.

[93] Kumar P., Agnihotri S., Roy I. (2016). Synthesis of dox drug conjugation and citric acid stabilized superparamagnetic iron-oxide nanoparticles for drug delivery. Biochem. Physiol..

[94] Lassenberger A., Scheber L.A., Stadlbauer A., Stiglbauer A., Helbich T., Reimhult E. (2017). Individually stabilised, superparamagnetic nanoparticles with a controlled shell and size, which exhibit exceptional stealth properties and high relaxivities. ACS Appl. Mater. Inter..

[95] Kohzadi S., Najmoddin N., Baharifar H., Shabani M. (2022). Functionalized SPION immobilized on graphene oxide: A study was conducted to investigate the potential anti-cancer and antiviral properties of the material. Diam. Relat. Mater..

[96] Uthaman S., Lee S.J., Cherukula K., Cho C.S., Park I.K. (2014). Polysaccharide-coated magnetic nanoparticles for imaging and gene therapy. Biomed. Res. Int..

[97] Karaagac O., Kockar H. (2022). Improvement of the saturation magnetization of PEG coated superparamagnetic iron oxide nanoparticles. J. Magn. Magn. Mater..

[98] Illes E., Szekeres M., Toth I.Y., Szabo A., Ivan B., Turcu R., Vekas L., Zupko I., Jaics G., Tombacz E. (2018). Multifunctional PEG-carboxylate copolymer coated superparamagnetic iron oxide nanoparticles for biomedical application. J. Magn. Magn. Mater..

[99] Hedayatnasab Z., Dabbagh A., Abnisa F., Daud W.M.A.W. (2020). Polycaprolactone-coated superparamagnetic iron oxide nanoparticles for in vitro magnetic hyperthermia therapy of cancer. Eur. Polym. J..

[100] Karimzadeh I., Aghazadeh M., Ganjali M.R., Norouzi P., Shirvani-Arani S., Doroudi T., Kolivand P.H., Marashi S.A., Gharailou D. (2016). A novel method for preparation of bare and poly(vinylpyrrolidone) coated superparamagnetic iron oxide nanoparticles for biomedical applications. Mater. Lett..

[101] Sagir T., Huysal M., Senel M., Isık S., Burgucu N., Tabakoglu O., Zaim M. (2022). Folic acid conjugated PAMAM-modified mesoporous silica-coated superparamagnetic iron oxide nanoparticles for potential cancer therapy. J. Colloid Interf. Sci..

[102] Chee H.L., Gan C.R.R., Ng M., Low L., Fernig D.G., Bhakoo K.K., Paramelle D. (2018). Biocompatible peptide-coated ultrasmall superparamagnetic iron oxide nanoparticles for in vivo contrast-enhanced magnetic resonance imaging. ACS Nano.

[103] Wani T.U., Raza S.N., Khan N.A. (2020). Nanoparticle opsonization: The forces involved and the protection afforded by long-chain polymers. Polym. Bull..

[104] Wallyn J., Anton N., Vandamme T.F. (2019). Synthesis, Principles, and Properties of Magnetite Nanoparticles for In Vivo Imaging Applications-A Review. Pharmaceutics.

[105] Jin R., Lin B., Li D., Ai H. (2014). Superparamagnetic iron oxide nanoparticles for MR imaging and therapy: Design considerations and clinical applications. Curr. Opin. Pharmacol..

[106] Ameen S., Fatima R., Ullah N., Tighezza A.M., Ali I., Bilal U., Saleem S., Bilal A.S.S. (2024). Investigation of structural, morphological, thermal, optical, and magnetic properties of graphene-embedded hematite and magnetite nanocomposites. Opt. Quant. Electron..

[107] Wahajuddin, Arora S. (2012). Superparamagnetic iron oxide nanoparticles: Magnetic nanoplatforms as drug carriers. Int. J. Nanomed..

[108] Zhang H., Li S., Liu Y., Yu Y., Lin S., Wang Q., Miao L., Wei H., Sun W. (2020). Fe_3_O_4_@GO Magnetic Nanocomposites Protect Mesenchymal Stem Cells and Promote Osteogenic Differentiation of Rat Bone Marrow Mesenchymal Stem Cells. Biomater. Sci..

[109] Krzyminiewski R., Kubiak T., Dobosz B., Schroeder G., Kurczewska J. (2014). EPR spectroscopy and imaging of TEMPO-labeled magnetite nanoparticles. Curr. Appl. Phys..

[110] Roessler M.M., Salvadori E. (2018). Principles and applications of EPR spectroscopy in the chemical sciences. Chem. Soc. Rev..

[111] Swapnalin J., Kumar S., Naidu K.C.B., Banerjee P. (2023). Recent Developments in Electron Paramagnetic Resonance for Spectroscopic Applications. Biointerface Res. Appl. Chem..

[112] Kubiak T. (2024). The Influence of Blood and Serum Microenvironment on Spin-Labeled Magnetic Nanoparticles. Magnetism.

[113] Han H., Li J., Santos H.A. (2023). Recent Advances in Fenton and Fenton-Like Reaction Mediated Nanoparticle in Cancer Therapy. Biomed. Technol..

[114] Nel A., Xia T., Madler L., Li N. (2006). Toxic Potential of Materials at the Nanolevel. Science.

[115] Xie Y., Liu D., Cai C., Chen X., Zhou Y., Wu L., Sun Y., Dai H., Kong X., Liu P. (2016). Size-Dependent Cytotoxicity of Fe_3_O_4_ Nanoparticles Induced by Biphasic Regulation of Oxidative Stress in Different Human Hepatoma Cells. Int. J. Nanomed..

[116] Zhang S., Wu S., Shen Y., Xiao Y., Gao L., Shi S. (2020). Cytotoxicity Studies of Fe_3_O_4_ Nanoparticles in Chicken Macrophage Cells. Roy. Soc. Open Sci..

[117] Yang L., Kuang H., Zhang W., Aguilar Z.P., Xiong Y., Lai W., Xu H., Wei H. (2015). Size-Dependent Biodistribution and Toxicokinetics of Iron Oxide Magnetic Nanoparticles in Mice. Nanoscale.

[118] Zhang J., Rana S., Srivastava R.S., Misra R.D.K. (2008). On the Chemical Synthesis and Drug Delivery Response of Folate Receptor-Activated, Polyethylene Glycol-Functionalized Magnetite Nanoparticles. Acta Biomater..

[119] Hu F., MacRenaris K.W., Waters E.A., Liang T., Schultz-Sikma E.A., Eckermann A.L., Meade T.J. (2009). Ultrasmall, Water-Soluble Magnetite Nanoparticles with High Relaxivity for Magnetic Resonance Imaging. J. Phys. Chem. C.

[120] Qiu Y., Wang Z., Owens A.C.E., Kulaots I., Chen Y., Kanec A.B., Hurt R.H. (2014). Antioxidant Chemistry of Graphene-Based Materials and Its Role in Oxidation Protection Technology. Nanoscale.

[121] Guo X., Mei N. (2014). Assessment of the Toxic Potential of Graphene Family Nanomaterials. J. Food Drug Anal..

[122] Hassani S., Gharehaghaji N., Divband B. (2022). Chitosan-coated iron oxide/graphene quantum dots as a potential multifunctional nanohybrid for bimodal magnetic resonance/fluorescence imaging and 5-fluorouracil delivery. Mater. Today Commun..

[123] Swain A.K., Pradhan L., Bahadur D. (2015). Polymer stabilized Fe_3_O_4_graphene as an amphiphilic drug carrier for thermo-chemotherapy of cancer. ACS Appl. Mater. Inter..

[124] Chen W., Wen X., Zhen G., Zheng X. (2015). Assembly of Fe_3_O_4_ nanoparticles on PEG-functionalized graphene oxide for efficient magnetic imaging and drug delivery. RSC Adv..

[125] Ma X., Tao H., Yang K., Feng L., Cheng L., Shi X., Li Y., Guo L., Liu Z. (2012). A Functionalized Graphene Oxide–Iron Oxide Nanocomposite for Magnetically Targeted Drug Delivery, Photothermal Therapy, and Magnetic Resonance Imaging. Nano Res..

[126] Song M.-M., Xu H.-L., Liang J.-X., Xiang H.-H., Liu R., Shen Y.-X. (2017). Lactoferrin modified graphene oxide iron oxide nanocomposite for glioma-targeted drug delivery. Mater. Sci. Eng. C.

[127] Fan X., Jiao G., Zhao W., Jina P., Li X. (2013). Magnetic Fe_3_O_4_–graphene composites as targeted drug nanocarriers for pH-activated release. Nanoscale.

[128] Aliabadia M., Shagholani H., Lehi A.Y. (2017). Synthesis of a novel biocompatible nanocomposite of graphene oxide and magnetic nanoparticles for drug delivery. Int. J. Biol. Macromol..

[129] Taheri-Kafrani A., Shirzadfar H., Kajani A.A., Kudhair B.K., Mohammed L.J., Mohammadi S., Lotfi F. (2021). Functionalized graphene oxide/Fe_3_O_4_ nanocomposite: A biocompatible and robust nanocarrier for targeted delivery and release of anticancer agents. J. Biotechnol..

[130] Karimi S., Namazi H. (2021). Fe_3_O_4_ @PEG-coated dendrimer modified graphene oxide nanocomposite as a pH-sensitive drug carrier for targeted delivery of doxorubicin. J. Alloy. Compd..

[131] Zhang R., Qin X., Kong F., Chen P., Pan G. (2019). Improving cellular uptake of therapeutic entities through interaction with components of cell membrane. Drug Deliv..

[132] Mohammadi Z.A., Aghamiri S.F., Zarrabi A., Talaie M.R. (2015). A comparative study on non-covalent functionalization of carbon nanotubes by chitosan and its derivatives for delivery of doxorubicin. Chem. Phys. Lett..

[133] Falank C., Tasset A.W., Farrell M., Harris S., Everill P., Marinkovic M., Reagan M.R. (2019). Development of medical-grade, discrete, multi-walled carbon nanotubes as drug delivery molecules to enhance the treatment of hematological malignancies. Nanomed. Nanotechnol..

[134] Mehdipoor E., Adeli M., Bavadi M., Sasanpour P., Rashidian B. (2011). A possible anticancer drug delivery system based on carbon nanotube–dendrimer hybrid nanomaterials. J. Mater. Chem..

[135] Cote B., Rao D., Alany R.G., Kwon G.S., Alani A.W.G. (2019). Lymphatic changes in cancer and drug delivery to the lymphatics in solid tumors. Adv. Drug Deliv. Rev..

[136] Caffo M., Curcio A., Rajiv K., Caruso G., Venza M., Germanò A. (2023). Potential role of carbon nanomaterials in the treatment of malignant brain gliomas. Cancers.

[137] Abskharoun S.B., Shawakfeh K.Q., Albiss B.A.A., Alsharaeh E.H. (2020). Magnetic Based Graphene Composites with Steroidal Diamine Dimer as Potential Drug in Hyperthermia Cancer Therapy. Mater. Res. Express.

[138] Laurent S., Dutz S., Häfeli U.O., Mahmoudi M. (2011). Magnetic Fluid Hyperthermia: Focus on Superparamagnetic Iron Oxide Nanoparticles. Adv. Colloid Interfac..

[139] Bobo D., Robinson K.J., Islam J., Thurecht K.J., Corrie S.R. (2016). Nanoparticle-Based Medicines: A Review of FDA-Approved Materials and Clinical Trials to Date. Pharm. Res. Dordr.

[140] Anselmo A.C., Mitragotri S. (2019). Nanoparticles in the Clinic: An Update. Bioeng. Transl. Med..

[141] Yang Y., Huang M., Qian J., Gao D., Liang X. (2020). Tunable Fe_3_O_4_ Nanorods for Enhanced Magnetic Hyperthermia Performance. Sci. Rep..

[142] Yusefi M., Shameli K., Yee O.S., Teow S.-Y., Hedayatnasab Z., Jahangirian H., Webster T.J., Kuča K. (2021). Green Synthesis of Fe_3_O_4_ Nanoparticles Stabilized by a Garcinia mangostana Fruit Peel Extract for Hyperthermia and Anticancer Activities. Int. J. Nanomed..

[143] Gu Y., Yoshikiyo M., Namai A., Bonvin D., Martinez A., Pinol R., Téllez P., Silva N.J.O., Ahrentorp F., Johansson C. (2020). Magnetic Hyperthermia with γ-Fe_2_O_3_ Nanoparticles. RSC Adv..

[144] Karade V.C., Parit S.B., Dawkar V.V., Devan R.S., Choudhary R.J., Kedge V.V., Pawar N.V., Kim J.H., Chougale A.D. (2019). A Green Approach for the Synthesis of α-Fe_2_O_3_ Nanoparticles from *Gardenia resinifera* Plant and Its In Vitro Hyperthermia Application. Heliyon.

[145] Gilchrist R.K., Medal R., Shorey W.D., Hanselman R.C., Parrott J.C., Taylor C.B. (1957). Selective Inductive Heating of Lymph Nodes. Ann. Surg..

[146] Hergt R., Dutz S. (2007). Magnetic Particle Hyperthermia-Biophysical Limitations of a Visionary Tumour Therapy. J. Magn. Magn. Mater..

[147] Chang D., Lim M., Goos J.A.C.M., Qiao R., Ng Y.Y., Mansfeld F.M., Jackson M., Davis T.P., Kavallaris M. (2018). Biologically Targeted Magnetic Hyperthermia: Potential and Limitations. Front. Pharmacol..

[148] Yang S.-J., Huang C.-H., Wang C.-H., Shieh M.-J., Chen K.-C. (2020). The Synergistic Effect of Hyperthermia and Chemotherapy in Magnetite Nanomedicine-Based Lung Cancer Treatment. Int. J. Nanomed..

[149] Issels R.D., Lindner L.H., Verweij J., Wessalowski R., Reichardt P., Wust P., Ghadjar P., Hohenberger P., Angele M., Salat C. (2018). Effect of Neoadjuvant Chemotherapy Plus Regional Hyperthermia on Long-Term Outcomes among Patients with Localized High-Risk Soft Tissue Sarcoma: The EORTC 62961-ESHO 95 Randomized Clinical Trial. JAMA Oncol..

[150] Arends T.J.H., Nativ O., Maffezzini A., de Cobelli O., Canepa G., Verweij B., Moskovitz A.G., van der Heijden J.A., Witjes J.A. (2016). Results of a Randomised Controlled Trial Comparing Intravesical Chemohyperthermia with Mitomycin C Versus Bacillus Calmette-Guérin for Adjuvant Treatment of Patients with Intermediate- and High-Risk Non–Muscle-Invasive Bladder Cancer. Eur. Urol..

[151] Liao C., Li Y., Tjong S.C. (2020). Polyetheretherketone and Its Composites for Bone Replacement and Regeneration. Polymers.

[152] Pei B., Wang W., Dunne N., Li X. (2019). Applications of Carbon Nanotubes in Bone Tissue Regeneration and Engineering: Superiority, Concerns, Current Advancements, and Prospects. Nanomaterials.

[153] Bozorgi A., Khazaei M., Soleimani M., Jamalpoor Z. (2021). Application of Nanoparticles in Bone Tissue Engineering: A Review on the Molecular Mechanisms Driving Osteogenesis. Biomater. Sci..

[154] Farjaminejad S., Farjaminejad R., Garcia-Godoy F. (2024). Nanoparticles in Bone Regeneration: A Narrative Review of Current Advances and Future Directions in Tissue Engineering. J. Funct. Biomater..

[155] Sanchez V.C., Jachak A., Hurt R.H., Kane A.B. (2012). Biological Interactions of Graphene-Family Nanomaterials: An Interdisciplinary Review. Chem. Res. Toxicol..

[156] Friedrich R.P., Cicha I., Alexiou C. (2021). Iron Oxide Nanoparticles in Regenerative Medicine and Tissue Engineering. Nanomaterials.

[157] Usui Y., Aoki K., Narita N., Murakami N., Nakamura I., Nakamura K., Ishigaki N., Yamazaki H., Horiuchi H., Kato H. (2008). Carbon Nanotubes with High Bone-Tissue Compatibility and Bone-Formation Acceleration Effects. Small.

[158] Narita N., Kobayashi Y., Nakamura H., Maeda K., Ishihara A., Mizoguchi T., Usui Y., Aoki K., Hara H., Kato H. (2009). Multiwalled Carbon Nanotubes Specifically Inhibit Osteoclast Differentiation and Function. Nano Lett..

[159] Shimizu M., Kobayashi Y., Mizoguchi T., Nakamura H., Kawahara I., Narita N., Usui Y., Aoki K., Hara K., Haniu H. (2012). Carbon Nanotubes Induce Bone Calcification by Bidirectional Interaction with Osteoblasts. Adv. Mater..

[160] Mukherjee S., Nandi S.K., Kundu B., Chanda A., Sen S., Das P.K. (2016). Enhanced Bone Regeneration with Carbon Nanotube Reinforced Hydroxyapatite in Animal Model. J. Mech. Behav. Biomed..

[161] Park J.E., Jang Y.S., Bae T.S., Lee M.H. (2019). Biocompatibility Characteristics of Titanium Coated with Multi Walled Carbon Nanotubes-Hydroxyapatite Nanocomposites. Materials.

[162] Abarrategi A., Gutierrez M.C., Moreno-Vicente C., Hortiguela M.J., Ramos V., Lopez-Lacomba J.L., Ferrer M.L., del Monte F. (2008). Multiwall Carbon Nanotube Scaffolds for Tissue Engineering Purposes. Biomaterials.

[163] Venkatesan J., Ryu B., Sudha P.N., Kim S.K. (2012). Preparation and Characterization of Chitosan-Carbon Nanotube Scaffolds for Bone Tissue Engineering. Int. J. Biol. Macromol..

[164] Chen L., Hu J., Shen X., Tong H. (2013). Synthesis and Characterization of Chitosan–Multiwalled Carbon Nanotubes/Hydroxyapatite Nanocomposites for Bone Tissue Engineering. J. Mater. Sci. Mater. Med..

[165] Tan W., Twomey J., Guo D., Madhavan K., Li M. (2010). Evaluation of nanostructural, mechanical, and biological properties of collagen-nanotube composites. IEEE T. Nanobiosci..

[166] Hirata E., Uo M., Takita H., Akasaka T., Watari F., Yokoyama A. (2011). Multiwalled carbon nanotube-coating of 3D collagen scaffolds for bone tissue engineering. Carbon.

[167] Jing Z., Wu Y., Su W., Tian M., Jiang W., Cao L., Zhao L., Zhao Z. (2017). Carbon Nanotube Reinforced Collagen/Hydroxyapatite Scaffolds Improve Bone Tissue Formation In Vitro and In Vivo. Ann. Biomed. Eng..

[168] Xu W., Ganz C., Weber U., Adam M., Holzhüter G., Wolter D., Frerich B., Vollmar B., Gerber T. (2011). Evaluation of injectable silica-embedded nanohydroxyapatite bone substitute in a rat tibia defect model. Int. J. Nanomed..

[169] Zhao J., Liu Y., Sun W.-B., Zhang H. (2011). Amorphous calcium phosphate and its application in dentistry. Chem. Cent. J..

[170] Kang M.S., Jeong S.J., Lee S.H., Kim B., Hong S.W., Lee J.H., Han D.-W. (2021). Reduced graphene oxide coating enhances osteogenic differentiation of human mesenchymal stem cells on Ti surfaces. Biomater. Res..

[171] Bellet P., Gasparotto M., Pressi S., Fortunato A., Scapin G., Mba M., Menna E., Filippini F. (2021). Graphene-Based Scaffolds for Regenerative Medicine. Nanomaterials.

[172] Zhang G., Zhen C., Yang J., Wang J., Wang S., Fang Y., Shan P. (2024). Recent advances of nanoparticles on bone tissue engineering and bone cells. Nanoscale Adv..

[173] Russo A., Bianchi M., Sartori M., Boi M., Giavaresi G., Salter D.M., Jelic M., Maltarellor M.C., Ortolani A., Sprio S. (2017). Bone regeneration in a rabbit critical femoral defect by means of magnetic hydroxyapatite microporous scaffolds. J. Biomed. Mater. Res. B.

[174] Demina A.M., Mekhaev A.V., Kandarakov O.F., Popenko V.I., Leonova O.G., Murzakaev A.M., Kuznetsov D.K., Uimin M.A., Minin A.S., Shur V.Y. (2020). L-Lysine-modified Fe_3_O_4_ nanoparticles for magnetic cell labeling. Colloid Surf. B.

[175] Marycz K., Sobierajska K., Komicka-Garbowska K., Kepska M., Idczak R., Nedelec J.M., Wiglusz R.J. (2020). Iron oxide nanoparticles (IOs) exposed to magnetic field promote expression of osteogenic markers in osteoblasts through integrin alpha-3 (INTa-3) activation, inhibits osteoclast activity and exerts anti-inflammatory action. J. Nanobiotechnol..

[176] Yu P., Zheng L., Wang P., Chai S., Zhang Y., Shi T., Zhang L., Peng R., Huang C., Guo B. (2020). Development of a novel polysaccharide-based iron oxide nanoparticle to prevent iron accumulation-related osteoporosis by scavenging reactive oxygen species. Int. J. Biol. Macromol..

[177] Babakhani A., Peighambardoust S.J., Olad A. (2024). Fabrication of magnetic nanocomposite scaffolds based on polyvinyl alcohol-chitosan containing hydroxyapatite and clay modified with graphene oxide: Evaluation of their properties for bone tissue engineering applications. J. Mech. Behav. Biomed. Mater..

[178] Panta P., Bergmann C. (2015). Raman spectroscopy of iron oxide nanoparticles. J. Mater. Sci. Eng..

[179] Besenhard M.O., LaGrow A.P., Hodzic A., Kriechbaum M., Panariello L., Bais G., Loizou K., Damilos S., Cruz M.M., Thanh N.T.H. (2020). Co-precipitation synthesis of stable iron oxide nanoparticles with NaOH: New insights and continuous production via flow chemistry. Chem. Eng. J..

[180] Logghe T., van Zwol E., Immordino B., Van den Cruys K., Peeters M., Giovannetti E., Bogers J. (2024). Hyperthermia in Combination with Emerging Targeted and Immunotherapies as a New Approach in Cancer Treatment. Cancers.

[181] Pérez-Herrero E., Lanier O.L., Krishnan N., D’Andrea A., Peppas N.A. (2024). Drug delivery methods for cancer immunotherapy. Drug Deliv. Transl. Re..

